# Exercise and depression symptoms in chronic kidney disease patients: an updated systematic review and meta-analysis

**DOI:** 10.1080/0886022X.2024.2436105

**Published:** 2024-12-03

**Authors:** Xueyi Zhou, Yan Bai, Fan Zhang, Min Gu

**Affiliations:** a Department of Nursing, Longhua Hospital Shanghai University of Traditional Chinese Medicine, Shanghai, China; b Department of Nephrology A, Longhua Hospital Shanghai University of Traditional Chinese Medicine, Shanghai, China

**Keywords:** Exercise, chronic kidney disease, depression symptom, systematic review, meta-analysis

## Abstract

**Objectives:**

To investigate whether exercise intervention is associated with reducing depressive symptoms in chronic kidney disease (CKD) patients.

**Methods:**

Medline (PubMed), Web of Science, Embase, and the Cochrane Central Register of Controlled Trials (CENTRAL) from inception to February 28, 2024. Randomized controlled trials comparing exercise intervention with usual care or stretching sessions for depression symptoms. Independent data extraction was conducted, and the quality of studies was assessed. A meta-analysis was carried out by using random effects models to calculate standardized mean difference (SMD) with a 95% confidence interval (95% CI) between groups.

**Results:**

23 trials with 1561 CKD patients were identified. Exercise interventions are associated with a significant reduction in depression symptoms among CKD patients, with a moderate average SMD of −0.726 (95% CI: −1.056, −0.396; *t*=-4.57; *p* < 0.001). Significant heterogeneity was observed (tau^2^ = 0.408 [95%CI: 0.227, 1.179], *I*^2^ = 79.9% [95% CI: 70.5%, 86.3%]). The funnel plot shows potential publication bias. Subgroup analyses showed that the beneficial effects of exercise on depression remained constant across all subgroups. The evidence is deemed as ‘very low’ certainty.

**Conclusions:**

Our systematic review and meta-analysis showed that exercise intervention was associated with significantly alleviating depression symptoms (certainty of evidence: very low). While the very low certainty of the evidence highlights a need for further research.

**PROSPERO registration number:**

CRD42021248450.

## 1. Introduction

Chronic kidney disease (CKD) is a global public health problem, affecting approximately 10–15% of the adult population worldwide [1]. CKD is characterized by a progressive loss of kidney function over time, leading to complications such as anemia, mineral and bone disorders, cardiovascular disease, and decreased quality of life [2]. Depression is a common comorbidity in CKD patients, with a prevalence rate of up to 30%, which is significantly higher than the general population [3]. The presence of depression in CKD patients is associated with increased morbidity, mortality, and healthcare utilization [4–6].

The etiology of depression in CKD patients is multifactorial, involving a complex interplay of biological, psychological, and social factors [7]. The chronic nature of the disease, the burden of treatment, and the associated lifestyle changes can contribute to the development of depression [8]. Additionally, the physiological changes associated with CKD, such as inflammation, oxidative stress, and neuroendocrine dysfunction, may play a role in the pathogenesis of depression [9].

Current treatment options for depression in CKD patients include pharmacological interventions, such as antidepressant medications, and psychological therapies, such as cognitive-behavioral therapy [10,11]. However, the use of antidepressants in this population may be limited by potential side effects and drug interactions, while access to psychological therapies may be restricted by availability and cost [12].

Exercise intervention has been recognized as a potential non-pharmacological approach to managing depression symptoms in various populations [13–16]. Exercise has been shown to have antidepressant effects through multiple mechanisms, including the release of endorphins, increased brain-derived neurotrophic factor, and improved self-efficacy [17]. In recent years, there has been growing interest in the potential benefits of exercise intervention for CKD patients with depression symptoms.

Previous meta-analyses have provided valuable insights into the association between exercise and depression symptoms in CKD patients [18,19]. However, these studies have limitations, such as inclusion of a limited number of studies, inadequate assessment of evidence, and absence of enough sensitivity analyses to confirm the robustness of the results. Given the mixed findings in the literature, there is a need to update systematic review and meta-analysis to synthesize the available evidence on the effects of exercise intervention on depression symptoms in CKD patients.

The purpose of this systematic review and meta-analysis is to evaluate the effects of exercise intervention on depression symptoms in CKD patients. We aim to address the following research questions:

1. What are the overall effects of exercise intervention on depression symptoms in CKD patients compared to control conditions?
2. Are there any differences in the effects of exercise intervention on depression symptoms based on the exercise type (e.g., aerobic, resistance, or combined), duration of intervention, or CKD stage?
3. What is the quality of the available evidence, and are there any limitations or gaps in the current literature?

## 2. Methods

### 2.1. Protocol and guidance

This study was conducted following Preferred Reporting Items for Systematic Reviews and Meta-Analysis (PRISMA 2020) [20]. The protocol for this review is registered with PROSPERO (CRD42021248450), which is like the current methodology with a few modifications (Table S1).

### 2.2. Inclusion and exclusion criteria

We considered as eligible trials those that: recruited adult (age ≥18 years) patients with chronic kidney disease, either predialysis or on renal replacement therapy; compared any form of exercise intervention (defined as planned, structured, and repetitive bodily movement done to improve and/or maintain one or more components of physical fitness[21]) with stretching sessions or usual care; provided scale-assessed depression scores. We restricted randomized controlled trials (RCT) published in English. If studies were case reports, observational studies, conference abstracts, and letters, we excluded them.

### 2.3. Search strategy

One of the authors (FZ) searched multiple databases: Medline (PubMed), Web of Science, Embase, and the Cochrane Central Register of Controlled Trials (CENTRAL) from inception to February 28, 2024. To maximize the search for relevant articles, we consulted reference lists of similar systematic reviews [18,19]. Details of the complete search strategy can be found in Table S2.

### 2.4. Study selection procedure

Search results were collated and duplicates were removed using Endnote 21.0 software. After removing duplicates, two independent researchers (XYZ and YB) read all titles and abstracts, followed by obtaining the full text of those that potentially met the eligibility criteria and further screening. We documented in detail the reasons for inclusion/exclusion of studies and the number of studies included and excluded at each stage. Disagreements, if any, were resolved by consensus.

### 2.5. Data extraction

Two independent researchers (XYZ and YB) extracted data from the included trials using standardized data tables: first author, year of publication, country, age, sample size, gender, disease stage, exercise type, and change values for depression scores. If a study mentioned an outcome of interest but did not provide an estimate, we contacted the authors to obtain the data. If there were disagreements, we resolved them by consensus.

If a study reported results from multiple time-point assessments, data from the last item were extracted. If a study reported data from two assessment tools [22], two authors discussed this issue and chose the tool that was most appropriate in terms of content for assessing depression in patients with CKD. If multiple interventions were reported in a trial (e.g., exercise training vs. dopamine vs. placebo intervention) [23], we included only the relevant measure (exercise training vs. placebo intervention). If multiple papers were published on the same participant, data were extracted from the study with the largest sample size to avoid duplication.

### 2.6. Risk of bias assessment

Two independent authors (XYZ and YB) assessed the risk of bias for each included RCT according to the Cochrane Collaboration’s Risk of Bias Tool 2 (RoB2), categorizing them as having a low risk of bias, some problems, or a high risk of bias [24]. A risk of bias summary table was generated using the Excel macro tool available on the official RoB2 website (https://www.riskofbias.info/welcome). Any disagreement about the methodologic quality assessment was resolved by consulting a third author.

### 2.7. Data synthesis

If a study did not provide change values for depression scores, the mean ± standard deviation (SD) of depression/anxiety scores at baseline (pre-intervention) and endpoint (post-intervention) were collected, and the change values were calculated by Formula A and Formula B. The SD was calculated according to Formula C and Formula D if the depression score was the mean and 95% confidence interval (CI). Where necessary, the mean ± SD was estimated from the median and interquartile range (Available at https://www.math.hkbu.edu.hk/∼tongt/papers/median2mean.html). If standard errors rather than SD were reported, standard errors were converted to SD using Formula E, where n is the number of subjects. For studies reporting subgroup results, Formula F was used for merging [25].

**Formula A:** ${\text{mean}}_{\text{change}}{\text{=mean}}_{\text{post‐intervention}}{\text{‐mean}}_{\text{baseline}}$
**Formula B:** ${\text{SD}}_{\text{change}}=\sqrt{{\text{SD}}_{\text{baseline}}^{2}+{\text{SD}}_{\text{postintervention}}^{2}-(2*R*{\text{SD}}_{\text{baseline}}*{\text{SD}}_{\text{postintervention}})}$
**Formula C:** (sample size of each group ≥100): $\text{SD}=\sqrt{n}*(\text{upper limit}-\text{lower limit})/3.92$
**Formula D:** (sample size of each group <60): $\text{SD}=\sqrt{n}*(\text{upper limit}-\text{lower limit})/(2*t)$
**Formula E:** SD= standard error ∗ √n
**Formula F:** ${\text{mean}}_{\text{combined}}=\frac{n_{1}m_{1}+n_{2}m_{2}}{n_{1}+n_{2}}$

### 2.8. Statistical analysis

Because depression scores are continuous variables that were assessed using inconsistent scales across studies, we used standardized mean difference (SMD) and 95% CI as summary statistics. Effect sizes were classified as negligible (0–0.2), small (>0.2–0.5), medium (>0.5–0.8) or large (>0.8). A primary meta-analysis was conducted that included RCTs comparing Δ in depression outcomes following an exercise intervention relative to control groups. This pooled meta-analysis had a total of seven separate subgroup analyses to describe the Δ in each depression outcome and to classify studies into subgroups based on the following factors: (1) age of participants in exercise group (<60 y vs. ≥60 y); (2) male proportion (<50% vs. ≥50%); (3) total sample (≤60 vs. >60); (4) intervention duration (≤12 weeks, >12 weeks); (5) exercise modality (aerobic exercise vs. resistance training vs. combined exercise; (6) disease stage (predialysis vs. peritoneal dialysis vs. hemodialysis); (7) region (Asia vs. Europe vs. North America vs. South America vs. Africa). Potential small study effects were assessed using funnel plots, and publication bias was examined using Begg rank correlation [26] and Egger regression tests [27]. In the case of funnel plot asymmetry, we further used contour-enhanced funnel plots to distinguish between funnel plot asymmetry caused by publication bias [28].

Statistical heterogeneity was assessed using *I*^2^ (<50%: low heterogeneity, 50–75%: moderate heterogeneity, and >75%: substantial heterogeneity). A random effects model was chosen to account for differences in true effect sizes across studies, given the expected differences between studies for different exercise intervention components and depression assessment tools. We estimated the heterogeneity parameter using restricted maximum likelihood and presented the 95% CIs using the Knapp-Hartung method.

We performed a series of sensitivity analyses to assess the robustness of the results of the meta-analysis: (1) to assess the impact of potential outliers, after excluding outlier studies and recalculating the pooled SMDs; (2) because the Consolidated Standards of Reporting Trials (CONSORT) statement corresponding to the RCT was published in 2010 [29], and studies published in earlier periods may have underreported and exaggerated results, we combined only RCTs from 2010 and later; (3) sensitivity analyses were also performed by comparing the SMDs of studies with and without imputed data (i.e., data extracted from figures or mean ± SD calculated from the median, interquartile range, or range); and (4) the leave-one-out method, in which one study at a time was removed to assess the impact of each study on the overall effect size.

Finally, meta-regressions were conducted to examine whether study characteristics explained the observed heterogeneity. Variables that served as predictors of study heterogeneity included age of participants in exercise group, male proportion, duration of intervention, total sample, exercise type, publication year, and disease stage.

All statistical analyses were performed in *R* using the *meta* [30] and *demtar* [31] packages. All statistical tests were two-sided and *P* values less than 0.05 were considered statistically significant.

### 2.9. Certainty of evidence

Two authors (YB and XYZ) independently assessed the certainty of evidence for depression scores from meta-analyses of pooled RCTs using the Grading of Recommendations Assessment, Development, and Evaluation (GRADE) method, with any conflicts resolved by a third author [32]. Certainty of evidence was graded from ‘high’ to ‘moderate’, ‘low’, or ‘very low’. GRADE certainty of evidence was downgraded if one or more of the following criteria were present: (1) risk of bias; (2) inconsistent; (3) indirectness; (4) imprecision; and (5) small study effect.

## 3. Results

### 3.1. Search results

**Figure 1.**
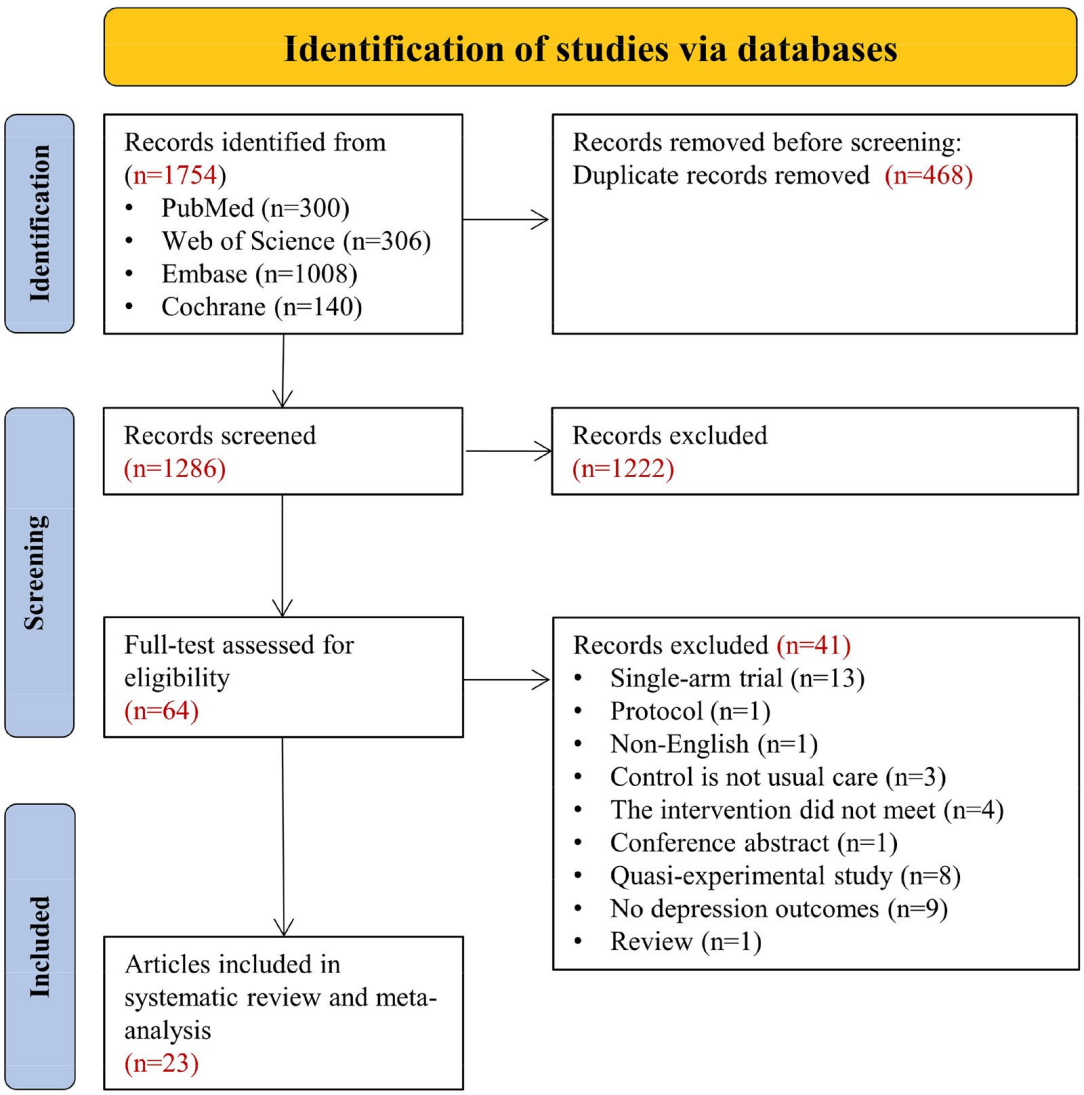
PRISMA Flow diagram. PRISMA, preferred reporting items for systematic reviews and meta-analyses.

After removing duplicate articles, an initial database search identified 1286 articles. Following a screening of titles and abstracts, an additional 1222 articles were removed. Based on the inclusion and exclusion criteria, the remaining 64 articles were selected for full-text review, of which 41 did not meet the inclusion criteria (Figure 1) (The reasons for full-text exclusion are listed in Table S3). Of these, a total of 23 eligible studies were included in this systematic review and meta-analysis [22,23,33–53].

### 3.2. Study characteristics

Table S4 presents participant demographic characteristics and changes in depression scores for each RCT. A total of 1561 participants were included in this systematic review, including 795 in the exercise group and 766 in the control group. The percentage of males ranged from 39.8% to 85.5%, and the mean age ranged from 38.8 to 66.1 years. Of the 23 RCTs included, 19 studies recruited hemodialysis-dependent CKD patients, three were predialysis patients, and only one was a peritoneal dialysis-dependent CKD patient, with studies from North America, South America, Europe, Asia, and Africa. Ten studies used the Beck Depression Inventory or Beck Depression Inventory-II, five studies used the Zung Depression Scale, four studies used the Center for Epidemiologic Studies-Depression Scale, two studies used the Hospital Anxiety and Depression scale, and one study each used the General Health Questionnaire-28 and the Geriatric Depression Scale to assess depressive symptoms. Risk assessments for the included literature are shown in Figure S1, and there were some concerns in all studies due to the specificity of exercise interventions themselves and the subjective nature of scale self-reporting. Tables S4 and S5 illustrate the estimates from the study by Carney and Zhao et al.

### 3.3. Main analysis

**Figure 2.**
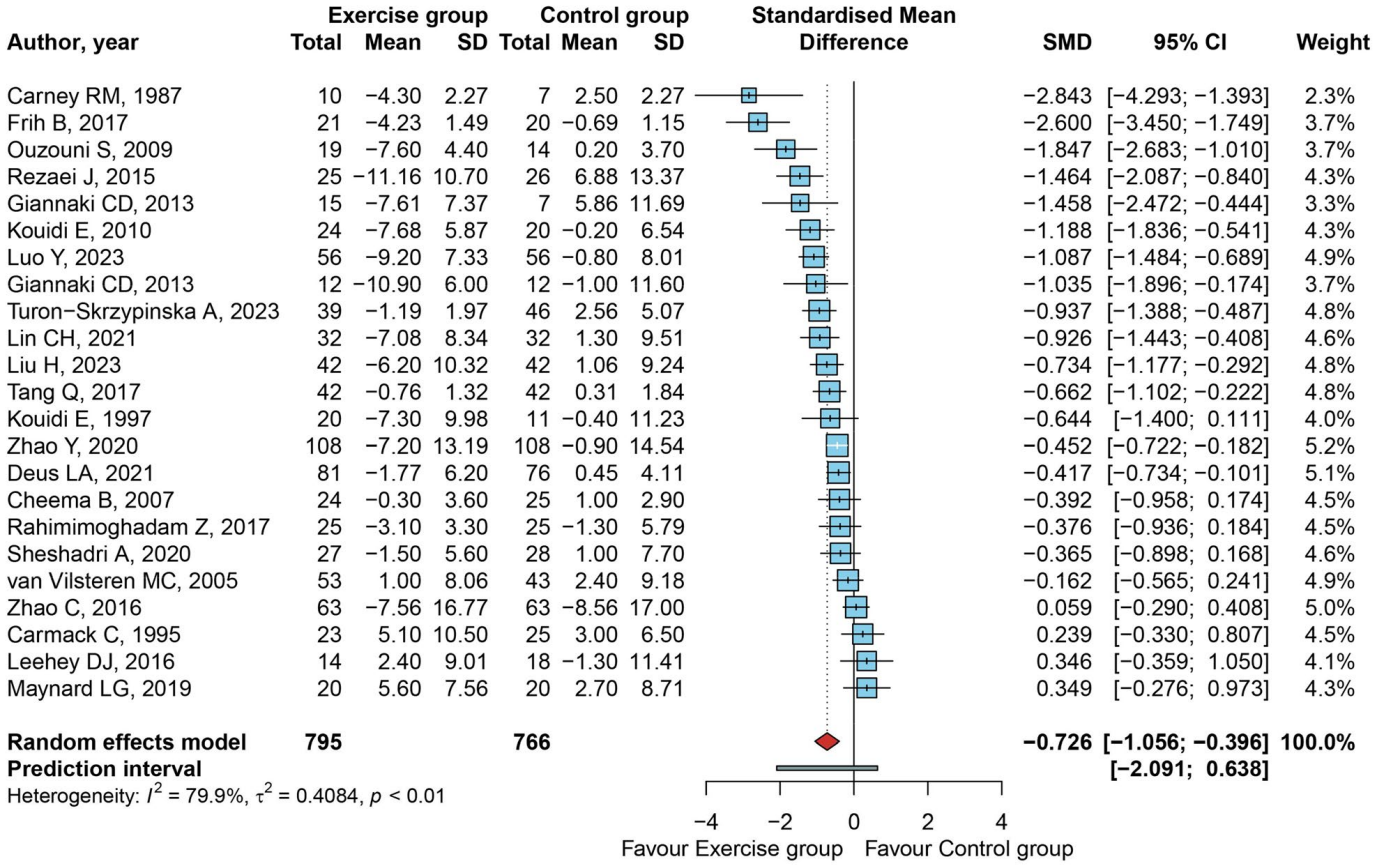
Forest plot of SMD (95% CI) showing the effectiveness of exercise intervention on depression symptoms.

The paired meta-analysis showed (Figure 2) that 19 (82.6%) out of 23 effects were less than zero, indicating that exercise favored reduction of depressive symptoms. There were 14 (60.9%) effects that significantly favored exercise intervention. The overall mean effect size was −0.726 (95% CI: −1.056, −0.396; *t*=-4.57; *p* < 0.001). The between-study heterogeneity variance was estimated at tau^2 = 0.408 (95%CI: 0.227, 1.179), with an *I*^2^ value of 79.9% (95%CI: 70.5%, 86.3%). The prediction interval ranged from SMD = −2.091 to 0.638, indicating that negative intervention effects cannot be ruled out for future studies.

### 3.4. Subgroup analyses

**Figure 3.**
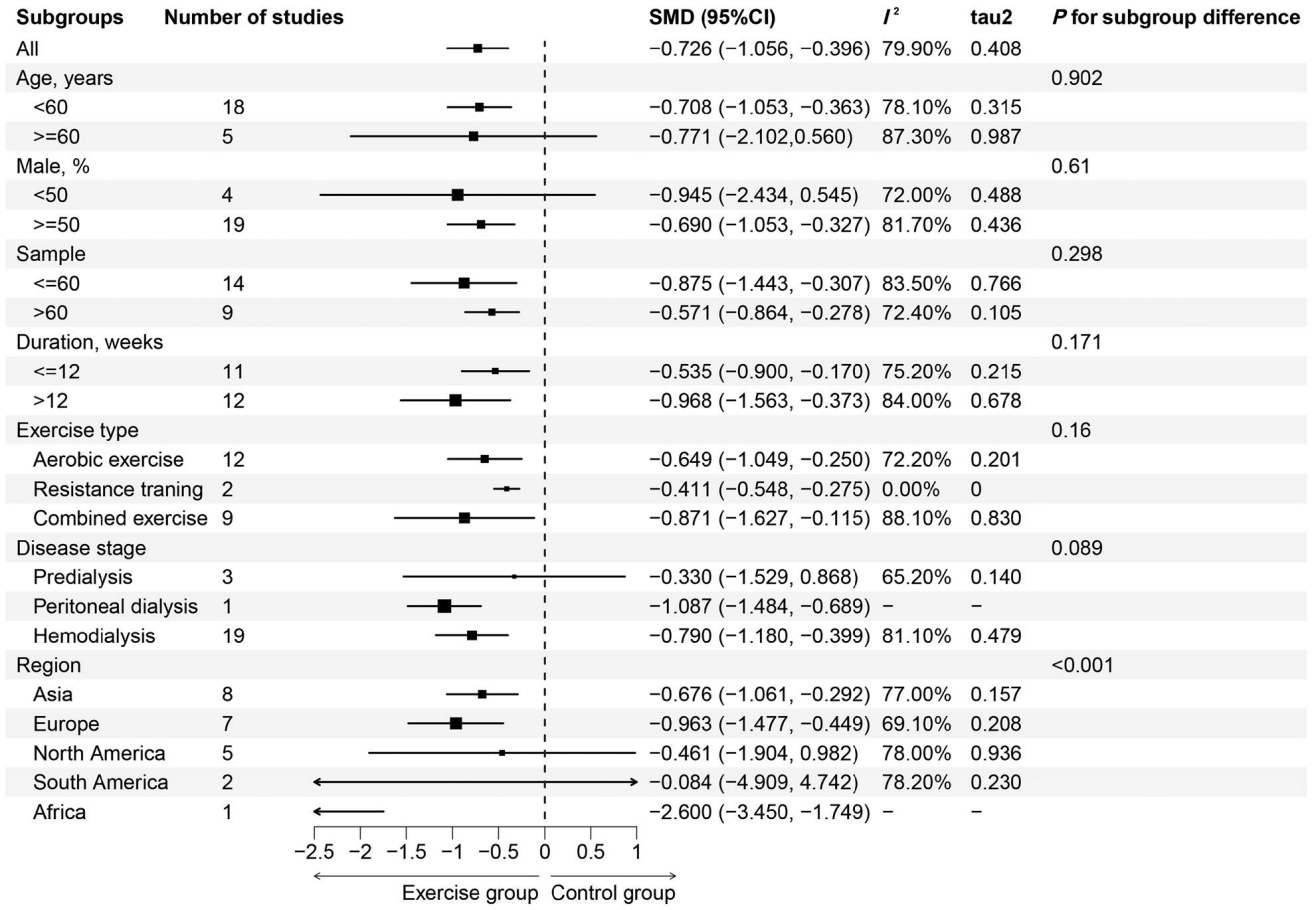
Subgroup meta-analysis of studies included in the quantitative analyses.

Subgroup analyses showed that the beneficial effects of exercise on depression remained constant across all subgroups in terms of age, male proportion, trial sample size, exercise duration, exercise type, disease stage, and region (Figure 3). Aerobic exercise (SMD=-0.649) and combined aerobic and resistance training (SMD=-0.871) showed a medium or large effect, while resistance training showed a small effect (SMD=-0.411). Larger effects were also found in studies of longer duration (SMD=-0.968), while shorter studies showed moderate effects (SMD=-0.535). For patients with different stages of CKD, exercise interventions appeared to be more effective for depressive symptoms in dialysis-dependent CKD patients (SMD for hemodialysis=-0.790; SMD for peritoneal dialysis=-1.087). Although the effect was greater in older patients (SMD=-0.771), it was not statistically significant, while younger showed a significant medium effect (SMD=-0.708).

### 3.5. Sensitivity analyses

After identifying six outlier studies (‘Maynard LG et al. [44]’, ‘Carney RM et al. [34]’, ‘Zhao C et al. [52]’, ‘Leehey DJ et al. [40]’, ‘Frih B et al. [37]’, ‘Carmack C et al. [33]’) using the *find.outliers* function, the combined results of the remaining 17 trials also reported a medium effect size (SMD: −0.754; 95% CI: −0.977, −0.531), but there was still moderate heterogeneity (*I*^2^=60.2%; 95% CI: 32.2%, 76.6%) (Figure S2).

Excluding 4 studies that estimated depression scores, the pooled analysis of the remaining 19 studies also reported a medium effect size (SMD: −0.640; 95% CI: −0.922 to −0.357) (Figure S3). The combined results for studies published after 2010 were SMD: −0.693 (95% CI: −1.066 to −0.320) (Figure S4). A leave-one-out sensitivity analysis showed stable results, with effect sizes ranging from −0.638 (95% CI: −0.929 to −0.346) to −0.771 (95% CI: −1.098 to −0.443) (Figure S5).

### 3.6. Publication bias

The Begg test (*z*=-2.19; *p* = 0.028) and Egger test (*t*=-2.31; *p* = 0.031) suggest that this meta-analysis may be subject to small-study effects. After adjusting for potential publication bias using the ‘trim and fill’ correction method, results similar to the overall pooled effect size estimate were obtained (Figure S6).

### 3.7. Meta-regression

When examining heterogeneity by meta-regression, the effect size of exercise interventions on depression symptoms in patients with CKD was not associated with age of participants in exercise group, male proportion, duration of intervention, total sample, exercise type, publication year, and disease stage (Table S7).

### 3.8. GRADE assessment

The evidence on exercise interventions on depression symptoms in patients with CKD was rated very low according to GRADE (Table 1), as most of the included studies had methodological flaws. In addition, meta-analysis results yielded substantial heterogeneity (*I*^2^=79.9%).

**Table 1.** GRADE evidence profile.

Certainty assessment	
NO. of studies	23
Study design	Randomized control trials
Risk of bias	Serious^a^
Inconsistency	Very serious^b^
Indirectness	Not serious^c^
Imprecision	Not serious^d^
Publication bias	Serious^e^
NO. of participants	
Participants	1561
Exercise intervention vs. Control group	795 vs. 766
Effect	
Absolute (95% CI)	−0.726(95% CI −1.056, −0.396)
Certainty	⨁◯◯◯
Importance	Critical

^a^ Despite the specificity of exercise interventions and subjective report outcomes, some studies still have a high risk of bias. Downgrade by one level.

^b^ Substantial heterogeneity due to *I*^2^=79.9%. Downgrade by two levels.

^c^ All studies were conducted in randomized controlled trials based on the CKD population. Not downgraded.

^d^ The 95% CI excludes the null value. Not downgraded.

^e^ This meta-analysis may be subject to small-study effects. Downgrade by one level.

## 4. Discussion

### 4.1. Principal findings

In this meta-analysis of 23 randomized controlled trials, involving 1561 participants, our findings suggest a moderate association between incorporating exercise interventions into clinical practice and alleviating depression symptoms in CKD patients, based on low certainty evidence. The observed SMD of −0.756 with a 95% CI of −1.067 to −0.446 indicates a statistically significant reduction in depression symptoms among CKD patients who underwent exercise interventions compared to those who did not, and this evidence is of ‘very low’ certainty. Subgroup analyses of the combined meta-analysis showed no differences in the improvement of depression symptoms in terms of types of exercise and duration of intervention.

These findings are consistent with previous studies that have investigated the effects of exercise on depression symptoms in various populations. A meta-analysis by Bellón et al. [13] found that exercise was associated with a significant reduction in depression symptoms among individuals without clinical depression. Similarly, a systematic review by Schuch et al. [16] concluded that exercise had significant antidepressant effects in people with major depressive disorder. A recent network meta-analysis of 218 unique studies has also shown that exercise is a ‘powerful weapon’ against depression [15].

However, it is important to note that there was significant heterogeneity in this study (*I*^2^ = 79.9%), which high heterogeneity may stem from several factors, including (1) differences in the characteristics of the populations included in our study (e.g., CKD stage, age); (2) heterogeneity of the exercise intervention protocols (type, intensity, frequency, duration); and (3) diversity of depression assessment tools. This high degree of heterogeneity suggests the need for caution in interpreting the results and underscores the need for more standardized intervention protocols and assessment tools in future studies.

The risk of bias assessment results for all included studies in this meta-analysis were problematic to some degree, which may have affected the reliability of our pooled estimates. The main sources of bias included the open nature of the exercise intervention (which could not be blinded) and the fact that assessment of depressive symptoms relied mostly on self-report. These factors may have contributed to the overestimation of effect sizes. For example, participants who knew they were receiving an exercise intervention may have reported more positive improvements in their own condition. We recognize that these limitations may affect the interpretation of results, and future studies should adopt more rigorous methodological designs, such as using objective depression assessment tools (e.g., clinical interviews or biomarkers) and longer follow-up periods, to improve the reliability of the evidence. Nonetheless, given the potential benefits and relative safety of exercise interventions, we believe that these findings still provide valuable insights into the management of depressive symptoms in CKD patients.

In addition, our analyses revealed the possibility of publication bias. Although we used a trim-and-fill method to make adjustments, publication bias may have led to an overestimation of the effect size. Therefore, we suggest that readers should be cautious in interpreting the results and urge future studies to be published regardless of the results to minimize publication bias.

### 4.2. Comparison with other studies

In the context of CKD patients, Hargrove et al. [19] pooled three randomized controlled trials using the Beck Depression Inventory and found that exercise interventions reduced depression scores by 35–40%, but the study was limited to hemodialysis-dependent CKD patients in 2021. In the same year, a meta-analysis by Ferreira et al. [18] showed that exercise was beneficial in reducing depressive symptoms compared to either active control (SMD = −0.66 [95% CI: −1.00, −0.33]) or passive control (mean difference = −6.95 [95% CI: −8.76, −5.14]). Our results further extend this key evidence due to different selection criteria and newly published trials. However, it is important to note that the current meta-analysis found the evidence to be of low certainty, suggesting that further high-quality research is needed to confirm these findings. The low certainty may be attributed to factors such as small sample sizes, heterogeneity in study designs, and potential biases in the included studies.

The prediction intervals in this study ranged from −2.091 to 0.638, a wide range that reflects a high degree of inter-study heterogeneity and provides important insights for future research. The prediction intervals suggest that although the results of our meta-analysis indicate that exercise interventions have a positive effect on depressive symptoms in CKD patients, future studies may observe a wide range of effects from negative to positive. This finding highlights the complexity of the effects of exercise interventions on depressive symptoms in CKD patients. Prediction intervals spanning zero mean that in some cases, exercise interventions may not have the expected positive effect and may even have a slight negative impact. This variability may stem from multiple factors, such as patient characteristics (e.g., CKD stage, comorbidities), type and intensity of exercise intervention, duration of intervention, and differences in study design. Therefore, we suggest that future studies should explore in depth the factors that may contribute to the variability of this effect in order to optimize the exercise intervention regimen and maximize its positive impact on depressive symptoms in patients with CKD. Meanwhile, individualized exercise prescription should be considered in clinical practice to adapt intervention strategies to patients’ specific conditions to ensure safety and efficacy.

### 4.3. Potential mechanisms

The mechanisms underlying the beneficial effects of exercise intervention on depression symptoms in CKD patients are multifaceted and complex. One potential mechanism is the release of endorphins during exercise, which can improve mood and reduce feelings of depression [54]. Exercise also promotes the release of brain-derived neurotrophic factor, a protein that supports the growth and survival of neurons in the brain, which may contribute to the antidepressant effects of exercise [17]. Furthermore, exercise can help reduce inflammation, a common feature in CKD patients that has been linked to depression [55]. Regular physical activity has been shown to decrease levels of pro-inflammatory cytokines, such as interleukin-6 and tumor necrosis factor-alpha, which may help alleviate depressive symptoms [56]. Third, exercise intervention can also improve sleep quality, which is often impaired in CKD patients and is associated with an increased risk of depression [57]. By promoting better sleep, exercise may help reduce depressive symptoms in this population [58]. Moreover, engaging in exercise can enhance self-efficacy and self-esteem, which are important factors in managing depression [59]. As CKD patients participate in exercise programs and experience improvements in their physical functioning, they may develop a greater sense of control over their health and well-being, leading to reduced depressive symptoms [60].

### 4.4. Public health implications

The findings of this meta-analysis have important public health implications for management of depression symptoms in CKD patients.

Given the low certainty of evidence supporting the effectiveness of exercise interventions in reducing depression symptoms among CKD patients, healthcare providers should cautiously consider incorporating exercise programs into the clinical management of this population. Exercise interventions may serve as an adjunct to traditional pharmacological and psychological treatments for depression, potentially reducing the need for antidepressant medications and their associated side effects [61].

Implementing exercise interventions in CKD patients may also have additional public health benefits beyond improving depression symptoms. Regular physical activity has been shown to improve cardiovascular health, physical functioning, and overall quality of life in CKD patients [62,63]. By promoting exercise as a means to manage depression symptoms, healthcare providers can simultaneously encourage the adoption of healthier lifestyles and potentially reduce the burden of other comorbidities in this population.

However, the low certainty of evidence highlights the need for further research to inform public health policies and clinical practice guidelines. Healthcare providers should consider the potential benefits and limitations of exercise interventions when making treatment decisions for CKD patients with depression, while also advocating for the development of evidence-based, patient-centered approaches to managing this important comorbidity. In addition, future studies should focus on determining the optimal type, intensity, duration, and frequency of exercise needed to achieve clinically meaningful improvements in depression symptoms [64].

The present study assessed the evidence’s certainty (‘very low’) using GRADE methodology, a rating that reflects multiple limitations of the current study, including high heterogeneity, risk of bias, and small-sample effects. The low certainty of the evidence implies that we lack sufficient confidence in the true effect of exercise interventions on depressive symptoms in patients with CKD, and that future research may significantly alter our perceptions. This finding has important implications for clinical practice and policy development. Although the results of the meta-analysis suggest that exercise interventions may be beneficial, we need to be more cautious before applying them widely in clinical practice. Healthcare professionals should fully consider individual differences and communicate adequately with patients when recommending exercise interventions to CKD patients.

### 4.5. Suggestions for future RCTs

Future RCTs investigating the effects of exercise intervention on depression symptoms in CKD patients should consider the following suggestions to improve the quality and generalizability of the evidence: (1) standardized exercise protocols: future RCTs should use standardized exercise protocols (e.g., Consensus on Exercise Reporting Template) [65], including the type, intensity, duration, and frequency of exercise, to allow for better comparisons across studies and to facilitate the implementation of effective interventions in clinical practice. (2) Long-term follow-up: studies should include longer follow-up periods to assess the sustainability of the effects of exercise intervention on depression symptoms and to evaluate any potential long-term benefits or adverse events. (3) Objective measures of depression: in addition to self-reported questionnaires, future RCTs should include objective measures of depression, such as clinical interviews or biomarkers, to provide a more comprehensive assessment of the intervention’s effects. Recently, Fedor et al. explored the use of wearable digital health technologies in the diagnosis, monitoring, and treatment of depression [66]. (4) Larger sample sizes: to improve statistical power and reduce the risk of type II errors, future RCTs should aim to recruit larger sample sizes based on appropriate power calculations. (5) Diverse populations: future studies should include more diverse CKD populations, such as peritoneal dialysis, hemodialysis, and kidney transplant recipients.

### 4.6. Strengths and limitations

One of the strengths of this study is that we followed the PRISMA guidelines and used a pre-registered protocol to ensure a transparent and reproducible process. In addition, we conducted various sensitivity analyses and subgroup analyses to confirm the robustness of the results. However, there are also several limitations to consider. Firstly, in some studies, depression is not the primary outcome, making it difficult to find such research because titles and abstracts may not necessarily describe depression assessment but rather present it as a secondary outcome in the paper. Despite rigorous screening processes, we cannot entirely rule out the possibility of some studies not being identified. Secondly, the included studies varied in terms of exercise protocols, participant characteristics, and outcome measures, which may have contributed to the heterogeneity observed in the meta-analysis. Thirdly, most of studies had relatively short follow-up periods, which limits our understanding of the long-term effects of exercise intervention on depression symptoms in CKD patients. Fourthly, asymmetric funnel plots may indicate publication bias, or they could be due to exaggeration of treatment effects in low-quality small studies. Sixth, the meta-analysis included studies predominantly focusing on CKD patients dependent on hemodialysis, which limits the generalizability of the findings to such a population. Seventh, the small sample effect of inclusion is also an important factor to consider. Small sample studies may overestimate the effect of an intervention, leading to an overestimation of the overall effect size. This emphasizes the importance of large samples and high-quality studies. Eighth, because this meta-analysis performed multiple subgroup analyses, there is a risk of increased Type I error. Future studies should consider correcting for multiple comparisons.

## 5. Conclusion

In conclusion, this systematic review and meta-analysis suggest that exercise interventions may be a promising approach for reducing depression symptoms in patients with CKD (certainty of evidence: very low). While the very low certainty of the evidence highlights a need for further research, the potential benefits of exercise for both mental and physical health in this population should not be overlooked. Integrating exercise into the comprehensive care of CKD patients may improve depression outcomes, enhance quality of life, and ultimately, contribute to better overall health and well-being.

## Supplementary Material

Supplemental Material

## Data Availability

Data are available upon reasonable request.
