# Supplementary material for: Exercise and depression symptoms in chronic kidney disease patients: an updated systematic review and meta-analysis
Source: Ren Fail. 2024 Dec 3;46(2):2436105. doi: 10.1080/0886022X.2024.2436105 (PMC11616742; doi:10.1080/0886022X.2024.2436105)

**Exercise and depression symptoms in chronic kidney disease patients: a updated systematic review and meta-analysis**

**Supplementary Material**

**Content**

[Table S1 Protocol deviations. 2](#_Toc164363432)

[Table S2 Search detailed for databases. 3](#_Toc164363433)

[Table S3 List of studies excluded at full-text review and reasons for exclusion. 8](#_Toc164363434)

[Table S4 Characteristics of randomized controlled trials included in the meta-analysis. 12](#_Toc164363435)

[Table S5 Estimation of standard deviation for Carney et al. 16](#_Toc164363436)

[Table S6 Estimation of standard deviation for Zhao C et al. 17](#_Toc164363437)

[Table S7 Results of meta-regression. 18](#_Toc164363438)

[Figure S1 Risk of bias for including studies. 19](#_Toc164363439)

[Figure S2 Sensitivity analysis excluding "outliers studies" 20](#_Toc164363440)

[Figure S3 Sensitivity analysis excluding "study on data estimation". 21](#_Toc164363441)

[Figure S4 Sensitivity analysis excluding "studies published before 2010". 22](#_Toc164363442)

[Figure S5 Sensitivity analysis based on "leave one out" approach. 23](#_Toc164363443)

[Figure S6 Contour-enhanced funnel plot. 24](#_Toc164363444)

Table S1 Protocol deviations.

| **Section** | **Previous protocol** | **Publication** | **Reasons** |
| --- | --- | --- | --- |
| Title | Effect of exercise interventions for sleep quality in patients with chronic kidney disease: a systematic review and meta-analysis | Exercise and depression symptoms in chronic kidney disease patients: an updated systematic review and meta-analysis | We recognize that the two outcomes are different, but the methodologies involved are nearly identical |
| Author | Fan Zhang, Ying Zhang, Liuyan Huang, Huachun Zhang, Qiuzi Sun, Hui Wang | Fan Zhang, Ying Zhang, Yan Bai, Yiling Chen, Yifei Zhong, Yi Li | - |
| Main outcome | Sleep quality | Depression symptoms | We used a previously registered protocol, and although the results are different, the methodology involved is consistent. |
| Risk of bias (quality) assessment | The methodological quality of each included systematic reviews will be evaluated using the 16 domains described in PEDro scale. | Two independent authors assessed the risk of bias for each included RCTs according to the Cochrane Collaboration's Risk of Bias Tool 2 (RoB2). | RoB2 is more applicable to the methodological assessment of RCTs. |
| Strategy for data synthesis | Tests of heterogeneity and meta-analysis were performed using Stata 12.0. | All statistical analyses were performed in R software. | - |

Table S2 Search detailed for databases.

| NO. | Detail | Results |
| --- | --- | --- |
|  | PubMed |  |
| 1 | "Exercise Movement Techniques"[MeSH] OR "Exercise Therapy"[MeSH] OR "Exercise"[MeSH] OR "Exercise"[title/abstract] OR "Endurance training"[title/abstract] OR "Jogging"[Mesh] OR "Jogging"[title/abstract] OR "Swimming"[Mesh] OR "Swimming"[title/abstract] OR "Walking"[Mesh] OR "Walking"[Title/Abstract] OR "Resistance exercise"[Title/Abstract] OR "Resistance training"[Title/Abstract] OR "Muscle training"[Title/Abstract] OR "Pilates"[title/abstract] OR "Tai Ji"[Mesh] OR "Tai-ji"[Title/Abstract] OR "Tai Chi"[Title/Abstract] OR "Tai Ji Quan"[Title/Abstract] OR "Taiji"[Title/Abstract] OR "Taijiquan"[Title/Abstract] OR "T'ai Chi"[Title/Abstract] OR "Qigong"[Title/Abstract] OR "Baduanjin"[Title/Abstract] OR "Yoga"[Title/Abstract] OR "Breathing Exercises"[MeSH] OR "Respiratory Muscles"[MeSH] OR "Respiratory Muscle Training"[Title/Abstract] OR "Respiratory Muscle Exercise"[Title/Abstract] OR "Inspiratory Muscle Training"[Title/Abstract] OR "Inspiratory Muscle Exercise"[Title/Abstract] OR "Expiratory Muscle Training"[Title/Abstract] OR "Breathing Exercise"[Title/Abstract] OR "Breathing Training"[Title/Abstract] OR "Respiratory Exercise"[Title/Abstract] OR "Inspiratory Exercise"[Title/Abstract] OR "Respiratory Training"[Title/Abstract] OR "Inspiratory Training"[Title/Abstract] OR "Vibration"[MeSH] OR "whole body vibration" [Title/Abstract] or "whole-body vibration" [Title/Abstract] or "vibration exercise" [Title/Abstract] or "vibration training" [Title/Abstract] or "WBV" [Title/Abstract] or "vibration therapy" [Title/Abstract] or "constant frequency vibration"[Title/Abstract] or "whole-body sinusoidal vibration"[Title/Abstract] | 648868 |
| 2 | "Renal insufficiency, chronic"[MeSH] OR "Renal Insufficiency"[MeSH] OR "Renal Replacement Therapy"[MeSH] OR "Renal Insufficiency"[Title/Abstract] OR "Kidney Insufficiency"[Title/Abstract] OR "Kidney failure"[Title/Abstract] OR "Renal failure"[Title/Abstract] OR "Kidney disease"[Title/Abstract] OR "Renal disease"[Title/Abstract] OR "Predialysis"[Title/Abstract] OR "Pre-dialysis"[Title/Abstract] OR "End-Stage Kidney"[Title/Abstract] OR "End-Stage Renal"[Title/Abstract] OR "Endstage Kidney"[Title/Abstract] OR "Endstage Renal"[Title/Abstract] OR "Dialysis"[Title/Abstract] OR "Hemodialysis"[Title/Abstract] OR "Haemodialysis"[Title/Abstract] OR "Hemodiafiltration"[Title/Abstract] OR "Haemodiafiltration"[Title/Abstract] OR "Hemofiltration"[Title/Abstract] OR "Haemofiltration"[Title/Abstract] OR "Renal Transplantation"[Title/Abstract] OR "Kidney Grafting"[Title/Abstract] OR "Kidney Transplantation"[Title/Abstract] | 545710 |
| 3 | "Anxiety"[MeSH] OR "Anxiety"[Title/Abstract] OR "Depressive Disorder"[MeSH] OR "Depression"[MeSH] OR "Depression"[Title/Abstract] | 676334 |
| 4 | #1 AND #2 AND #3 | 300 |
| 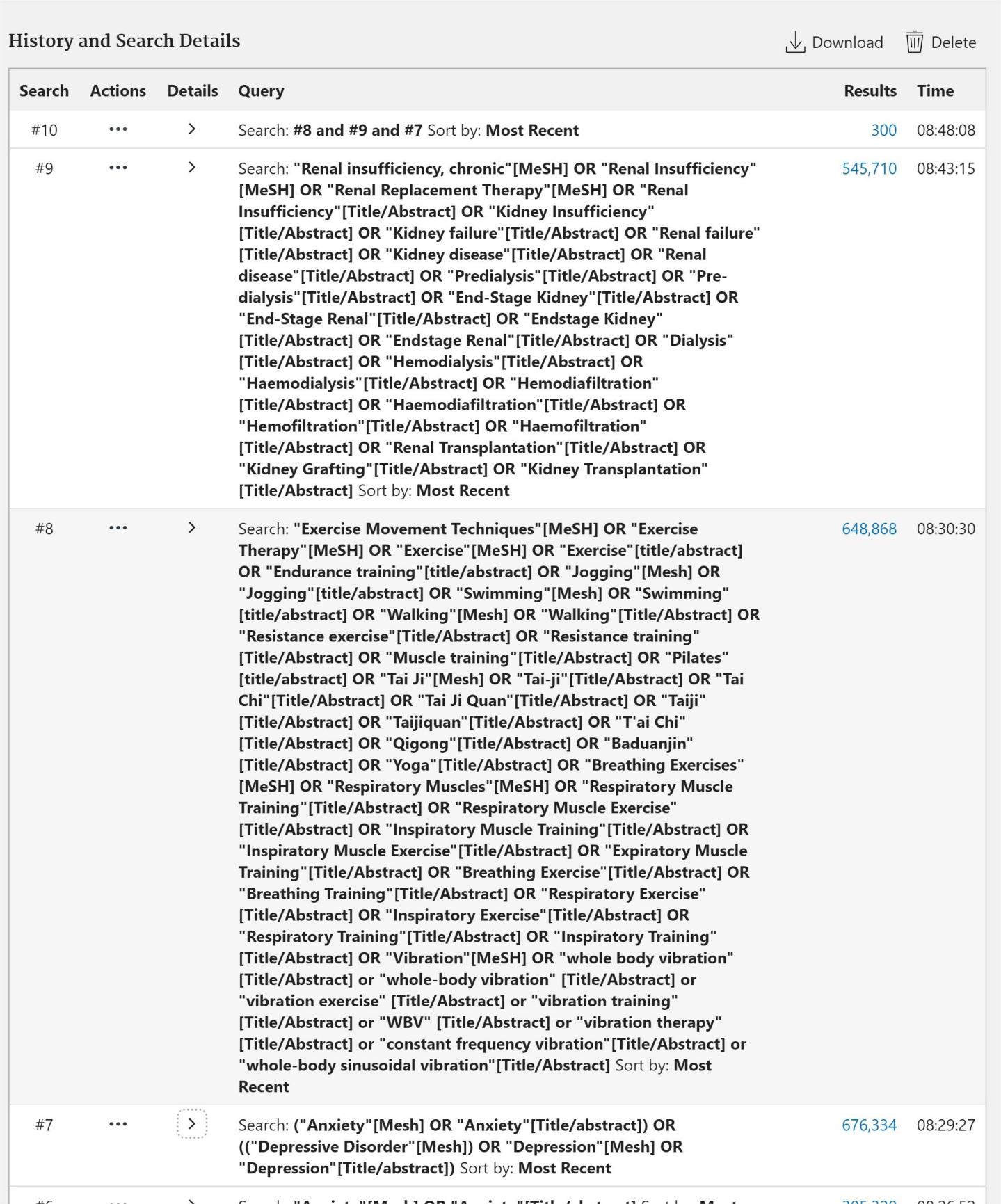 | | |
|  | Embase |  |
| 1 | 'exercise'/exp OR 'walking'/exp OR 'swimming'/exp OR 'Tai Chi'/exp OR 'resistance training'/exp OR 'yoga'/exp OR 'breathing exercise'/exp OR 'vibration'/exp OR 'Exercise':ti,ab,kw OR 'Endurance training':ti,ab,kw OR 'Jogging':ti,ab,kw OR 'Swimming':ti,ab,kw OR 'Walking':ti,ab,kw OR 'Resistance exercise':ti,ab,kw OR 'Resistance training':ti,ab,kw OR 'Muscle training':ti,ab,kw OR 'Pilates':ti,ab,kw OR 'Tai-ji':ti,ab,kw OR 'Tai Chi':ti,ab,kw OR 'Tai Ji Quan':ti,ab,kw OR 'Taiji':ti,ab,kw OR 'Taijiquan':ti,ab,kw OR 'Qigong':ti,ab,kw OR 'Baduanjin':ti,ab,kw OR 'Yoga':ti,ab,kw OR 'Respiratory Muscle Training':ti,ab,kw OR 'Respiratory Muscle Exercise':ti,ab,kw OR 'Inspiratory Muscle Training':ti,ab,kw OR 'Inspiratory Muscle Exercise':ti,ab,kw OR 'Expiratory Muscle Training':ti,ab,kw OR 'Breathing Exercise':ti,ab,kw OR 'Breathing Training':ti,ab,kw OR 'Respiratory Exercise':ti,ab,kw OR 'Inspiratory Exercise':ti,ab,kw OR 'Respiratory Training':ti,ab,kw OR 'Inspiratory Training':ti,ab,kw OR 'whole body vibration':ti,ab,kw OR 'whole-body vibration':ti,ab,kw OR 'vibration exercise':ti,ab,kw OR 'vibration training':ti,ab,kw OR 'vibration therapy':ti,ab,kw OR 'constant frequency vibration':ti,ab,kw OR 'whole-body sinusoidal vibration':ti,ab,kw | 938112 |
| 2 | 'kidney failure'/exp OR 'renal replacement therapy'/exp OR 'kidney transplantation'/exp OR 'Renal Replacement Therapy':ti,ab,kw OR 'Renal Insufficiency':ti,ab,kw OR 'Kidney Insufficiency':ti,ab,kw OR 'Kidney failure':ti,ab,kw OR 'Renal failure':ti,ab,kw OR 'Kidney disease':ti,ab,kw OR 'Renal disease':ti,ab,kw OR 'Predialysis':ti,ab,kw OR 'Pre-dialysis':ti,ab,kw OR 'End-Stage Kidney':ti,ab,kw OR 'End-Stage Renal':ti,ab,kw OR 'Endstage Kidney':ti,ab,kw OR 'Endstage Renal':ti,ab,kw OR 'Dialysis':ti,ab,kw OR 'Hemodialysis':ti,ab,kw OR 'Haemodialysis':ti,ab,kw OR 'Hemodiafiltration':ti,ab,kw OR 'Haemodiafiltration':ti,ab,kw OR 'Hemofiltration':ti,ab,kw OR 'Haemofiltration':ti,ab,kw OR 'Renal Transplantation':ti,ab,kw OR 'Kidney Grafting':ti,ab,kw OR 'Kidney Transplantation':ti,ab,kw | 973600 |
| 3 | 'anxiety'/exp OR 'anxiety':ti,ab,kw OR 'depression assessment'/exp OR 'depression'/exp OR 'depression':ti,ab,kw | 1141285 |
| 4 | #1 AND #2 AND #3 | 1008 |
| 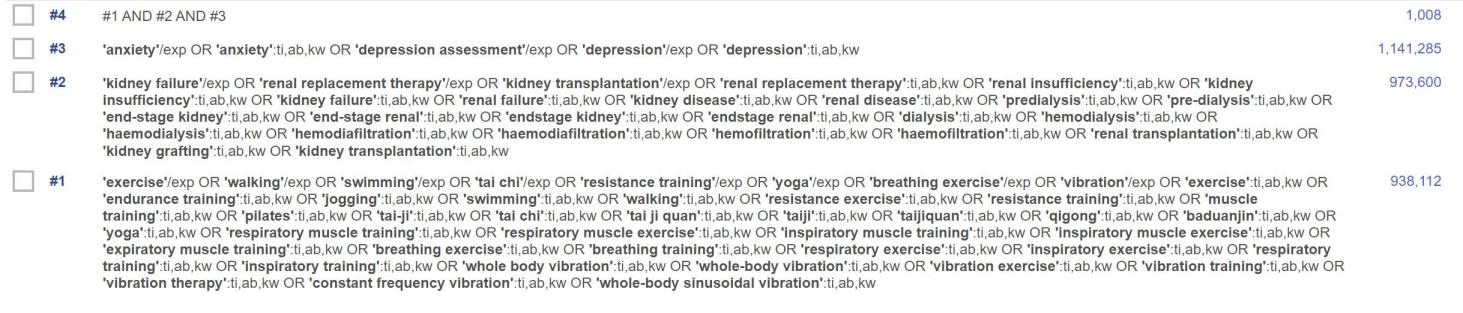 | | |
|  | Web of Science |  |
|  | TS=("Exercise" OR "Endurance training" OR "Jogging" OR "Swimming" OR "Walking" OR "Resistance exercise" OR "Resistance training" OR "Muscle training" OR "Pilates" OR "Tai-ji" OR "Tai Chi" OR "Tai Ji Quan" OR "Taiji" OR "Taijiquan" OR "T'ai Chi" OR "Baduanjin" OR "Qigong" OR "Yoga" OR "Respiratory Muscle Training" OR "Respiratory Muscle Exercise" OR "Inspiratory Muscle Training" OR "Inspiratory Muscle Exercise" OR "Expiratory Muscle Training" OR "Breathing Exercise" OR "Breathing Training" OR "Respiratory Exercise" OR "Inspiratory Exercise" OR "Respiratory Training" OR "Inspiratory Training" OR "whole body vibration" OR "whole-body vibration" OR "vibration exercise" OR "vibration training" OR "vibration therapy" OR "constant frequency vibration" OR "whole-body sinusoidal vibration") | 574292 |
|  | TS=("Renal Insufficiency" OR "Kidney Insufficiency" OR "Kidney failure" OR "Renal failure" OR "Kidney disease" OR "Renal disease" OR "Predialysis" OR "Pre-dialysis" OR "End-Stage Kidney" OR "End-Stage Renal" OR "Endstage Kidney" OR "Endstage Renal" OR "Dialysis" OR "Hemodialysis" OR "Haemodialysis" OR "Hemodiafiltration" OR "Haemodiafiltration" OR "Hemofiltration" OR "Haemofiltration" OR "Renal Transplantation" OR "Kidney Grafting" OR "Kidney Transplantation") | 467788 |
|  | TS=("depression" OR "anxiety") | 616197 |
| 4 | #1 AND #2 AND #3 | 306 |
| 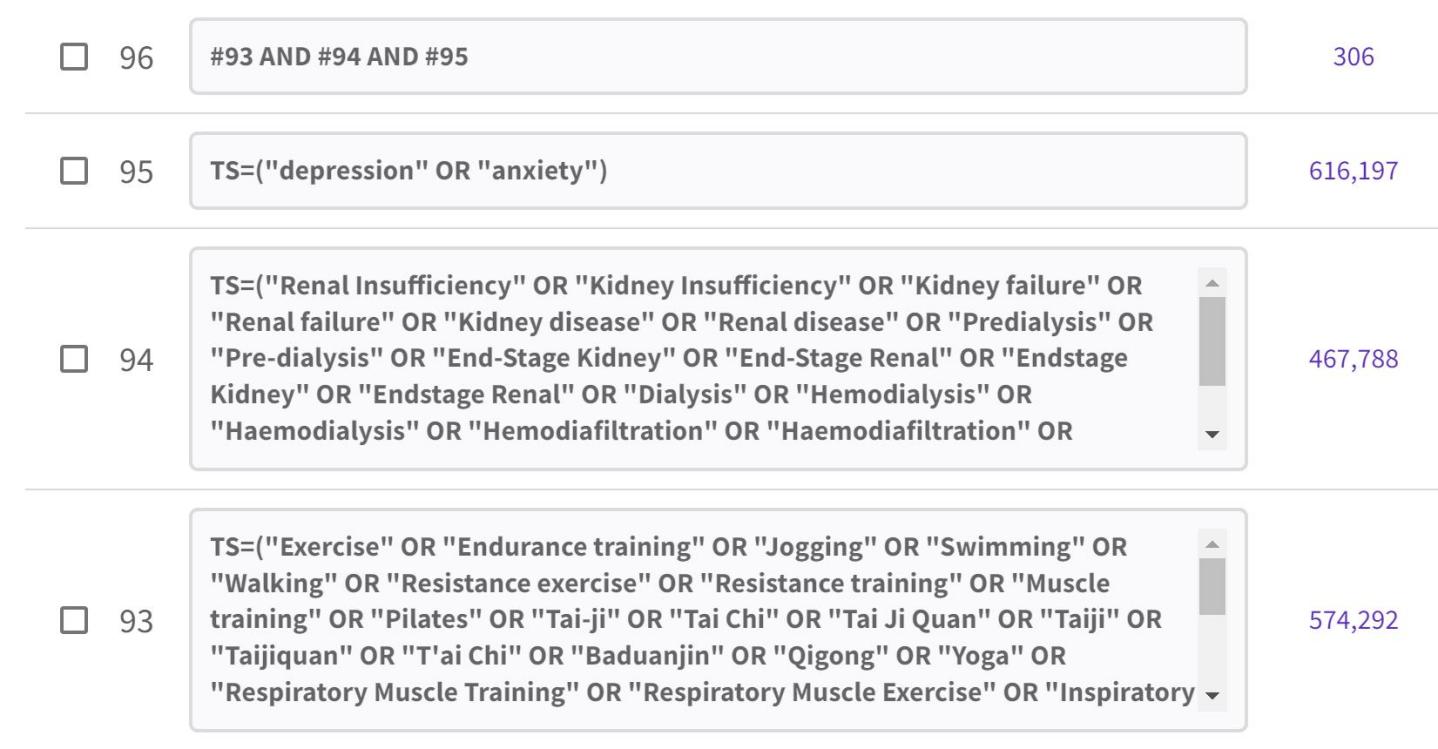 | | |
|  | Cochrane library |  |
| #1 | MeSH descriptor: [Exercise Movement Techniques] explode all trees | 3480 |
| #2 | MeSH descriptor: [Exercise Therapy] explode all trees | 21433 |
| #3 | MeSH descriptor: [Exercise] explode all trees | 38282 |
| #4 | ("Exercise" OR "Endurance training"):ti,ab,kw | 130945 |
| #5 | MeSH descriptor: [Jogging] explode all trees | 69 |
| #6 | MeSH descriptor: [Swimming] explode all trees | 648 |
| #7 | MeSH descriptor: [Walking] explode all trees | 8164 |
| #8 | MeSH descriptor: [Tai Ji] explode all trees | 572 |
| #9 | MeSH descriptor: [Breathing Exercises] explode all trees | 1331 |
| #10 | MeSH descriptor: [Respiratory Muscles] explode all trees | 1214 |
| #11 | MeSH descriptor: [Vibration] explode all trees | 1588 |
| #12 | ("Jogging" OR "Swimming" OR "Walking" OR "Resistance exercise" OR "Resistance training" OR "Muscle training" OR "Pilates" OR "Tai-ji" OR "Tai Chi" OR "Tai Ji Quan" OR "Taiji" OR "Taijiquan" OR "T'ai Chi" OR "Qigong" OR "Baduanjin" OR "Yoga" OR "Respiratory Muscle Training" OR "Respiratory Muscle Exercise" OR "Inspiratory Muscle Training" OR "Inspiratory Muscle Exercise" OR "Expiratory Muscle Training" OR "Breathing Exercise" OR "Breathing Training" OR "Respiratory Exercise" OR "Inspiratory Exercise" OR "Respiratory Training" OR "Inspiratory Training" OR "whole body vibration" or "whole-body vibration" or "vibration exercise" or "vibration training" or "WBV" or "vibration therapy" or "constant frequency vibration" or "whole-body sinusoidal vibration"):ti,ab,kw | 60707 |
| #13 | #1 OR #2 OR #3 OR #4 OR #5 OR #6 OR #7 OR #8 OR #9 OR #10 OR #11 OR #12 | 160814 |
| #14 | MeSH descriptor: [Renal insufficiency, chronic] explode all trees | 9583 |
| #15 | MeSH descriptor: [Renal Insufficiency] explode all trees | 12992 |
| #16 | MeSH descriptor: [Renal Replacement Therapy] explode all trees | 12572 |
| #17 | ("Renal Insufficiency" OR "Kidney Insufficiency" OR "Kidney failure" OR "Renal failure" OR "Kidney disease" OR "Renal disease" OR "Predialysis" OR "Pre-dialysis" OR "End-Stage Kidney" OR "End-Stage Renal" OR "Endstage Kidney" OR "Endstage Renal" OR "Dialysis" OR "Hemodialysis" OR "Haemodialysis" OR "Hemodiafiltration" OR "Haemodiafiltration" OR "Hemofiltration" OR "Haemofiltration" OR "Renal Transplantation" OR "Kidney Grafting" OR "Kidney Transplantation"):ti,ab,kw | 54937 |
| #18 | #14 OR #15 OR #16 OR #17 | 55747 |
| #19 | MeSH descriptor: [Anxiety] explode all trees | 12386 |
| #20 | MeSH descriptor: [Depressive Disorder] explode all trees | 16414 |
| #21 | MeSH descriptor: [Depression] explode all trees | 17908 |
| #22 | ("Anxiety" OR "Depression"):ti,ab,kw | 102430 |
| #23 | #19 OR #20 OR #21 OR #22 | 107830 |
| #24 | #13 AND #18 AND #23 in Trials | 140 |
| 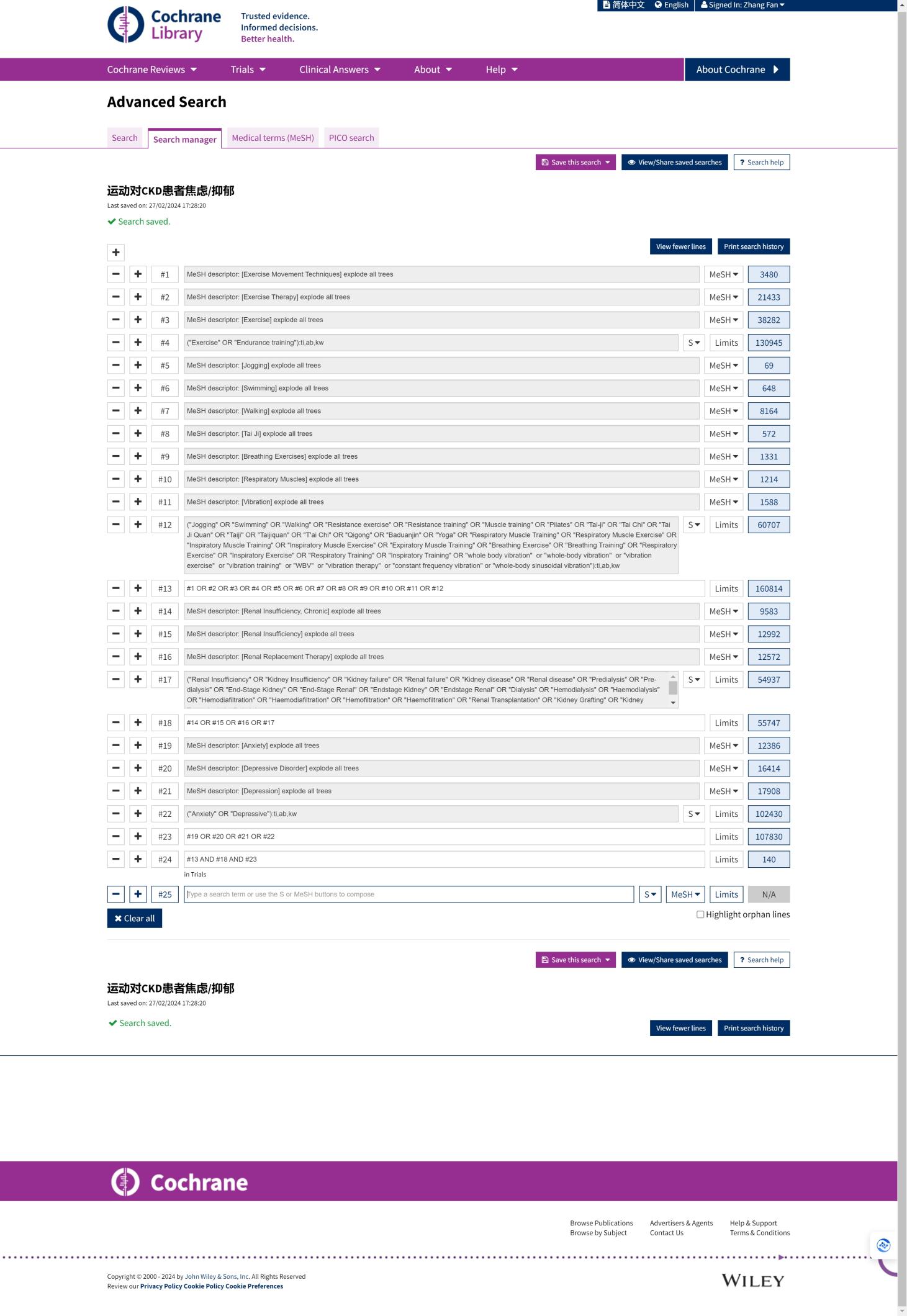 | | |

Table S3 List of studies excluded at full-text review and reasons for exclusion.

| **NO.** | **Reference** | **Reasons** |
| --- | --- | --- |
| 1 | M. Jhamb, S. M. Devaraj, M. Alemairi, et al. A Comprehensive Exercise (COMEX) Intervention to Optimize Exercise Participation for Improving Patient-Centered Outcomes and Physical Functioning in Patients Receiving Hemodialysis: Development and Pilot Testing. KIDNEY MED 2023;5(11):100720. doi: 10.1016/j.xkme.2023.100720 | Single-arm trial |
| 2 | F. Levendoğlu, L. Altintepe, N. Okudan, et al. A twelve week exercise program improves the psychological status, quality of life and work capacity in hemodialysis patients. J NEPHROL 2004;17(6):826-32. | Single-arm trial |
| 3 | L. Zhou, D. Shi, L. Zhang, et al. Does Intradialytic Group Exercise Programme Influence Patient-Reported Outcomes, Laboratory Parameters, and Anthropometric Parameters in Maintenance Hemodialysis Patients? A Single-Group Repeated-Measures Trial. PATIENT PREFER ADHER 2023;17:491-501. doi: 10.2147/PPA.S400005 | Single-arm trial |
| 4 | CSC Rosa, C. D. Giannaki, A. Krase, et al. Effects of 12 months of detraining on health-related quality of life in patients receiving hemodialysis therapy. INT UROL NEPHROL 2020;52(9):1771-78. doi: 10.1007/s11255-020-02560-5 | Single-arm trial |
| 5 | Y. M. Liu, Y. C. Chung, J. S. Chang, M. L. Yeh. Effects of Aerobic Exercise During Hemodialysis on Physical Functional Performance and Depression. BIOL RES NURS 2015;17(2):214-21. | Single-arm trial |
| 6 | M. R. Suh, H. H. Jung, S. B. Kim, J. S. Park, W. S. Yang. Effects of regular exercise on anxiety, depression, and quality of life in maintenance hemodialysis patients. RENAL FAILURE 2002;24(3):337-45. doi: 10.1081/jdi-120005367 | Single-arm trial |
| 7 | S. A. Greenwood, H. Lindup, K. Taylor, et al. Evaluation of a pragmatic exercise rehabilitation programme in chronic kidney disease. NEPHROL DIAL TRANSPL 2012;27 Suppl 3:iii126-34. doi: 10.1093/ndt/gfs272 | Single-arm trial |
| 8 | H. Parent-Roberge, T. A. Deshayes, C. Fortier, et al. Feasibility of an Intradialytic Combined Exercise Program Targeting Older Adults With End-Stage Renal Disease. J AGING PHYS ACTIV 2021;29(6):905-14. doi: 10.1123/japa.2020-0359 | Single-arm trial |
| 9 | S. Y. Rhee, J. K. Song, S. C. Hong, et al. Intradialytic exercise improves physical function and reduces intradialytic hypotension and depression in hemodialysis patients. KOREAN J INTERN MED 2019;34(3):588-98. doi: 10.3904/kjim.2017.020 | Single-arm trial |
| 10 | P. N. Bennett, T. Parsons, R. Ben-Moshe, et al. Intradialytic Laughter Yoga therapy for haemodialysis patients: a pre-post intervention feasibility study. BMC COMPLEM ALTERN M 2015;15:176. doi: 10.1186/s12906-015-0705-5 | Single-arm trial |
| 11 | S. S. Grigoriou, A. A. Krase, C. Karatzaferi, et al. Long-term intradialytic hybrid exercise training on fatigue symptoms in patients receiving hemodialysis therapy. INT UROL NEPHROL 2021;53(4):771-84. doi: 10.1007/s11255-020-02711-8 | Single-arm trial |
| 12 | G. Romano, R. Simonella, E. Falleti, et al. Physical training effects in renal transplant recipients. CLIN TRANSPLANT 2010;24(4):510-14. | Single-arm trial |
| 13 | Z. Kauric-Klein. The Effect of a Yoga Intervention on Physical and Psychological Outcomes in Patients on Chronic Hemodialysis. NEPHROL NURS J 2022;49(4):335-81. | Single-arm trial |
| 14 | P. N. Bennett, R. M. Daly, S. F. Fraser, et al. The impact of an exercise physiologist coordinated resistance exercise program on the physical function of people receiving hemodialysis: A stepped wedge randomised control study. BMC NEPHROL 2013;14(1) doi: 10.1186/1471-2369-14-204 | Protocol |
| 15 | Simo V. Esteve, Jiménez A. Junqué, Guzmán F. Moreno, et al. Benefits of a low intensity exercise programme during haemodialysis sessions in elderly patients. NEFROLOGIA 2015;35(4):385-94. doi: 10.1016/j.nefro.2015.03.006 | Non-English |
| 16 | L. O. P. De Villar, B. P. Domínguez, J. M. Gramage, et al. Comparison of intradialytic versus home-based exercise programmes on physical function, physical level and health related quality of life. NEPHROL DIAL TRANSPL 2016;31:i477-78. doi: 10.1093/ndt/gfw191 | Control is not usual care |
| 17 | E. Lendraitiene, E. Lanevskaite, D. Petrusevicienė, K. Berskienė, V. Lendraitis. Effect of Different Physical Therapy Programs on Renal Transplant Recipients' Physical Activity, Grip Strength, and Psychoemotional Status and the Associations Between These Indices. TRANSPL P 2018;50(10):3338-45. doi: 10.1016/j.transproceed.2018.07.009 | Control is not usual care |
| 18 | W. Dziubek, J. Kowalska, M. Kusztal, et al. The Level of Anxiety and Depression in Dialysis Patients Undertaking Regular Physical Exercise Training--a Preliminary Study. KIDNEY BLOOD PRESS R 2016;41(1):86-98. doi: 10.1159/000368548 | Control is not usual care |
| 19 | H. Eroglu, Z. G. Metin. Benson Relaxation Technique Combined With Music Therapy for Fatigue, Anxiety, and Depression in Hemodialysis Patients A Randomized Controlled Trial. HOLIST NURS PRACT 2022;36(3):139-48. doi: 10.1097/HNP.0000000000000509 | The intervention did not meet |
| 20 | S. H. Tsai, M. Y. Wang, N. F. Miao, et al. The Efficacy of a Nurse-Led Breathing Training Program in Reducing Depressive Symptoms in Patients on Hemodialysis: A Randomized Controlled Trial. AM J NURS 2015;115(4):24-42. doi: 10.1097/01.NAJ.0000463023.48226.16 | The intervention did not meet |
| 21 | B. Frih, W. Mkacher, A. Bouzguenda, et al. Effects of listening to Holy Qur’an recitation and physical training on dialysis efficacy, functional capacity, and psychosocial outcomes in elderly patients undergoing haemodialysis. LIBYAN J MED 2017;12(1) doi: 10.1080/19932820.2017.1372032 | The intervention did not meet |
| 22 | F. C. Willingham, I. Speelman, J. Hamilton, et al. Feasibility and effectiveness of pre-emptive rehabilitation in persons approaching dialysis (PREHAB). J RENAL CARE 2019;45(1):9-19. doi: 10.1111/jorc.12262 | The intervention did not meet |
| 23 | A. Kwon, F. Feldman, P. M. Leung, D. MacKey, T. Parsons. The effects of a pre-dialytic exercise program on the health of hemodialysis patients. HEMODIAL INT 2018;22(1):A13. | Conference abstract |
| 24 | H. Yabe, K. Kono, T. Yamaguchi, et al. Effect of intradialytic exercise on geriatric issues in older patients undergoing hemodialysis: a single-center non-randomized controlled study. INT UROL NEPHROL 2022;54(11):2939-48. doi: 10.1007/s11255-022-03205-5 | Quasi-experimental study |
| 25 | S. J. Moug, S. Grant, G. Creed, M. Boulton Jones. Exercise during haemodialysis: West of Scotland pilot study. SCOT MED J 2004;49(1):14-17. doi: 10.1177/003693300404900104 | Quasi-experimental study |
| 26 | R. Shalom, J. A. Blumenthal, R. S. Williams, R. G. McMurray, V. W. Dennis. Feasibility and benefits of exercise training in patients on maintenance dialysis. KIDNEY INT 1984;25(6):958-63. doi: 10.1038/ki.1984.117 | Quasi-experimental study |
| 27 | P. L. Valenzuela, A. de Alba, R. Pedrero-Chamizo, et al. Intradialytic exercise: One size doesn't fit all. FRONT PHYSIOL 2018;9(JUL) doi: 10.3389/fphys.2018.00844 | Quasi-experimental study |
| 28 | N. Nakamura-Taira, N. Horikawa, F. Oka, et al. Quasi-cluster randomized trial of a six-month low-intensity group-based resistance exercise for hemodialysis patients on depression and cognitive function: a 12-month follow-up. HEALTH PSYCHOL BEHAV 2021;9(1):741-60. doi: 10.1080/21642850.2021.1966302 | Quasi-experimental study |
| 29 | R. Barroso, A. C. Silva-Filho, C. J. Dias, et al. Effect of exercise training in heart rate variability, anxiety, depression, and sleep quality in kidney recipients: A preliminary study. J HEALTH PSYCHOL 2019;24(3):299-308. doi: 10.1177/1359105316676329 | Quasi-experimental study |
| 30 | E. A. Sanad, H. A. E. H. El-Shinnawy, H. A. Hebah, et al. Effect of intra-dialytic physical exercise on depression in prevalent hemodialysis patients. Egyptian Journal of Neurology, Psychiatry and Neurosurgery 2022;58(1) doi: 10.1186/s41983-022-00560-z | Quasi-experimental study |
| 31 | Y. M. Liu, Y. C. Chung, J. S. Chang, M. L. Yeh. Effects of Aerobic Exercise During Hemodialysis on Physical Functional Performance and Depression. BIOL RES NURS 2015;17(2):214-21. doi: 10.1177/1099800414539548 | Quasi-experimental study |
| 32 | Giannaki CD, Sakkas GK, Karatzaferi C, Maridaki MD, Koutedakis Y, Hadjigeorgiou GM, Stefanidis I. Combination of Exercise Training and Dopamine Agonists in Patients with RLS on Dialysis: A Randomized, Double-Blind Placebo-Controlled Study. ASAIO J. 2015 Nov-Dec;61(6):738-41. doi: 10.1097/MAT.0000000000000271. | No depression outcomes |
| 33 | G. S. Birdee, R. L. Rothman, S. J. Sohl, et al. Feasibility and Safety of Intradialysis Yoga and Education in Maintenance Hemodialysis Patients. J RENAL NUTR 2015;25(5):445-53. doi: 10.1053/j.jrn.2015.02.004 | No depression outcomes |
| 34 | Z. Rahimimoghadam, Z. Rahemi, Z. Sadat, N. M. Ajorpaz. Pilates exercises and quality of life of patients with chronic kidney disease. COMPLEMENT THER CLIN 2019;34:35-40. doi: 10.1016/j.ctcp.2018.10.017 | No depression outcomes |
| 35 | S. S. Grigoriou, C. Karatzaferi, C. D. Giannaki, et al. The effect of a 9-month hybrid intradialytic exercise training program on nerve conduction velocity parameters in patients receiving hemodialysis therapy. INT UROL NEPHROL 2022;54(12):3271-81. doi: 10.1007/s11255-022-03266-6 | No depression outcomes |
| 36 | P. Zhang, S. Liu, X. Zhu, et al. The effects of a physical exercise program in Chinese kidney transplant recipients: a prospective randomised controlled trial. CLIN KIDNEY J 2023;16(8):1316-29. doi: 10.1093/ckj/sfad065 | No depression outcomes |
| 37 | J. H. Cho, J. Y. Lee, S. Lee, et al. Effect of intradialytic exercise on daily physical activity and sleep quality in maintenance hemodialysis patients. INT UROL NEPHROL 2018;50(4):745-54. doi: 10.1007/s11255-018-1796-y | No depression outcomes |
| 38 | C. Y. Wu, H. M. Han, M. C. Huang, et al. Effect of qigong training on fatigue in haemodialysis patients: A non-randomized controlled trial. COMPLEMENT THER MED 2014;22(2):244-50. doi: 10.1016/j.ctim.2014.01.004 | No depression outcomes |
| 39 | F. Salehi, M. Dehghan, P. Mangolian Shahrbabaki, M. R. Ebadzadeh. Effectiveness of exercise on fatigue in hemodialysis patients: A randomized controlled trial. BMC Sports Science, Medicine and Rehabilitation 2020;12(1) doi: 10.1186/s13102-020-00165-0 | No depression outcomes |
| 40 | F. Moeinzadeh, S. Shahidi, S. Shahzeidi. Evaluating the effect of intradialytic cycling exercise on quality of life and recovery time in hemodialysis patients: A randomized clinical trial. J RES MED SCI 2022;27(1) doi: 10.4103/jrms.jrms_866_21 | No depression outcomes |
| 41 | H. Yu, M. Huang, Y. Tao, et al. The effects of exercise training interventions on depression in hemodialysis patients. FRONT PSYCHIATRY 2023;14:1321413. doi: 10.3389/fpsyt.2023.1321413 | Review |

Table S4 Characteristics of randomized controlled trials included in the meta-analysis.

| **Author** | **Year** | **Country** | **Sample (F/M)** | **Age** | **Stage** | **Intervention group** | **Control group** | **Duration** | **Depression assessment tool** | **Baseline depression score** | **Endpoint depression score** | **∆Change (endpoint-baseline)** |
| --- | --- | --- | --- | --- | --- | --- | --- | --- | --- | --- | --- | --- |
| Giannaki CD | 2013 | Greece | 24 (17/7) | I: 59.2±11.8 | HD | Aerobic exercise | Usual care | 24 weeks | Zung depression scale | I: 45.1±6.5 | I: 34.2±7.0 | I: -10.9±6.0 |
|  |  |  |  | C: 58.0±10.7 |  |  |  |  |  | C: 44.7±12.7 | C: 43.7±6.7 | C: -1.0±11.6 |
| Sheshadri A | 2020 | USA | 60 (47/13) | I: 60 (53-66) | HD | Aerobic exercise | Usual care | 12 weeks | Center for Epidemiologic Studies-Depression Scale | I: 9.0±11.9 | I: 11.3±12.4 | I: 1.5±5.6 |
|  |  |  |  | C: 56 (51-65) |  |  |  |  |  | C: 7.9±7.3 | C: 6.6±6.5 | C: -1.0±7.7 |
| Maynard LG | 2019 | Brazil | 40 (22/18) | I: 49±15.2 | HD | Combined exercise | Usual care | 12 weeks | Center for Epidemiologic Studies–Depression Scale | I: 12.7±7.8 | I: 7.1±7.3 | I: -5.60±7.56 |
|  |  |  |  | C: 43.9±11.7 |  |  |  |  |  | C: 15.8±7.8 | C: 13.1±9.4 | C: -2.7±8.71 |
| Tang Q | 2017 | China | 84 (51/33) | I: 46.26±15.61 | Predialysis | Aerobic exercise | Usual care | 12 weeks | Hospital Anxiety and Depression scale | I: 5.29±3.06 | I: 4.52±2.62 | I: -0.76±1.32 |
|  |  |  |  | C: 43.90±12.44 |  |  |  |  |  | C: 6.10±3.18 | C: 6.40±2.84 | C: 0.31±1.84 |
| Lin CH | 2021 | China | 64 (41/23) | I: 62.0±9.5 | HD | Aerobic exercise | Usual care | 12 weeks | Beck Depression Inventory | I: 12.08±9.3 | I: 5.0±6.8 | I: -7.08±8.34 |
|  |  |  |  | C: 62.1±12.3 |  |  |  |  |  | C: 11.2±9.8 | C: 12.5±9.2 | C: 1.3±9.51 |
| Ouzouni S | 2009 | Greece | 33 (27/6) | I: 47.4±15.7 | HD | Combined exercise | Usual care | 40 weeks | Beck Depression Inventory | I: 19.3±4.9 | I: 11.7±3.6 | I: -7.6±4.40 |
|  |  |  |  | C: 50.5±11.7 |  |  |  |  |  | C: 19.2±3.3 | C: 19.4±4.0 | C: 0.2±3.70 |
| Kouidi E | 1997 | Greece | 31 (15/16) | I: 49.6±12.1 | HD | Aerobic exercise | Usual care | 24 weeks | Beck Depression Inventory | I: 21.0±10.4 | I: 13.7±9.5 | I: -7.3±9.98 |
|  |  |  |  | C: 52.8±10.2 |  |  |  |  |  | C: 21.7±10.4 | C: 21.3±11.9 | C: -0.4±11.23 |
| Carney RM | 1987 | USA | 17 (8/9) | I: 36.1±3.2 | HD | Aerobic exercise | Usual | 24 weeks | Beck Depression Inventory | I: 7.7±1.9 |  | I: -4.3 |
|  |  |  |  | C: 40.7±5.3 |  |  |  |  |  | C: 7.2±1.3 |  | C: 2.5 ^1)^ |
| Turoń-Skrzypińska A | 2023 | Poland | 85 (58/27) | I: 57.56±17.61 | HD | Aerobic exercise | Usual care | 12 weeks | Beck Depression Inventory | I: 9.53±6.43 | I: 8.31±6.29 | I: -1.19±1.97 |
|  |  |  |  | C: 62.63±15.47 |  |  |  |  |  | C: 9.09±8.25 | C: 11.64±10.25 | C: 2.56±5.07 |
| Leehey DJ | 2016 | USA | 36 (18/18) | I: 65.4±8.7 | Predialysis | Combined exercise+diet management | Diet management | 52 weeks | Center for Epidemiologic Studies-Depression Scale | I: 12.7±8.7 | I: 10.3±9.3 | I: -2.4±9.01 |
|  |  |  |  | C: 66.6±7.5 |  |  |  |  |  | C: 15.4±9.4 | C: 16.7±12.7 | C: 1.3±11.41 |
| Frih B | 2017 | Tunisia | 41 (21/20) | I: 64.2±3.4 | HD | Combined exercise | Usual care | 16 weeks | Hospital Anxiety and Depression scale | I: 11.92±1.11 | I: 7.69±1.69 | I: -4.23±1.49 |
|  |  |  |  | C: 65.2±3.1 |  |  |  |  |  | C: 12.85±1.14 | C: 12.16±1.15 | C: -0.69±1.15 ^2)^ |
| van Vilsteren MC | 2005 | The Netherlands | 85 (64/21) | I: 52±15 | HD | Combined exercise | Usual care | 12 weeks | Zung depression scale | I: 36.2±7.8 | I: 37.2±8.3 | I: 1±8.06 |
|  |  |  |  | C: 58±16 |  |  |  |  |  | C: 39.0±8.7 | C: 41.4±9.6 | C: 2.4±9.18 |
| Liu H | 2023 | China | 84 (41/43) | I: 56.3±11.10 | HD | Aerobic exercise | Usual care | 24 weeks | Zung depression scale | I: 36.59±10.23 | I: 30.39±10.41 | I: -6.2±10.32 |
|  |  |  |  | C: 59.2±10.41 |  |  |  |  |  | C: 37.59±9.28 | C: 38.65±9.20 | C: 1.06±9.24 |
| Luo Y | 2023 | China | 112 (65/47) | I: 54.1±14.5 | PD | Combined exercise | Usual care | 12 weeks | Beck Depression Inventory-II | I: 24.8±7.7 | I: 15.6±6.9 | I: -9.2±7.33 |
|  |  |  |  | C: 53.8±15.2 |  |  |  |  |  | C: 23.5±8.2 | C: 22.7±7.8 | C: -0.8±8.01 |
| Giannaki CD | 2013 | Greece | 22 (16/6) | I: 56.4±12.5 | HD | Aerobic exercise | Usual care | 24 weeks | Zung depression scale | I: 43.45±8.06 | I: 35.84±6.38 | I: -7.61±7.37 |
|  |  |  |  | C: 56.8±16.5 |  |  |  |  |  | C: 37.85±12.15 | C: 43.71±11.17 | C: 5.86±11.69 |
| Zhao Y | 2020 | China | 216 (86/130) | I: 56.1±10.8 | Predialysis | Combined exercise | Usual care | 24 weeks | Zung depression scale | I: 30.0±15.0 | I: 22.8±9.8 | I: -7.2±13.19 |
|  |  |  |  | C: 58.1±9.7 |  |  |  |  |  | C: 30.1±15.1 | C: 29.2±13.9 | C: -0.9±14.54 ^3)^ |
| Rezaei J | 2015 | Iran | 51 (35/16) | I: 43.96±7.86 | HD | Combined exercise | Usual care | 10 weeks | Beck Depression Inventory | I: 23.80±10.29 | I: 12.64±11.07 | I: -11.16±10.70 |
|  |  |  |  | C: 42.61±12.67 |  |  |  |  |  | C: 19.23±12.98 | C: 26.11±13.72 | C: 6.88±13.37 |
| Kouidi E | 2010 | Greece | 44 (26/18) | I: 46.3±11.2 | HD | Combined exercise | Usual care | 48 weeks | Beck Depression Inventory | I: 22.29±6.71 | I: 14.61±4.15 | I: -7.68±5.87 |
|  |  |  |  | C: 45.8±10.9 |  |  |  |  |  | C: 22.30±6.81 | C: 22.10±6.24 | C: -0.2±6.54 ^4)^ |
| Deus LA | 2021 | Brazil | 157 (86/71) | I: 67.27±3.24 | HD | Resistance training | Usual care | 24 weeks | Beck Depression Inventory | I: 28.10±6.11 | I: 26.33±6.28 | I: -1.77±6.20 |
|  |  |  |  | C: 66.33±3.88 |  |  |  |  |  | C: 26.83±3.81 | C: 27.28±4.35 | C: 0.45±4.11 |
| Rahimimoghadam Z | 2017 | Iran | 50 (41/9) | I: 39.1±2.2 | HD | Aerobic exercise | Usual care | 8 weeks | General Health Questionnaire-28 | I: 11.7±3.5 | I: 8.6±3.06 | I: -3.1±3.3 |
|  |  |  |  | C: 38.4±1.8 |  |  |  |  |  | C: 11.7±6.6 | C: 10.4±2.4 | C: -1.3±5.79 |
| Cheema B | 2007 | USA | 49 (34/15) | I: 60.0±15.3 | HD | Resistance training | Usual care | 12 weeks | Geriatric Depression Scale | I: 57.7±22.2 | I: | I: -0.3±3.6 |
|  |  |  |  | C: 65.0±12.9 |  |  |  |  |  | C: 55.8±23.7 | C: | C: 1.0±2.9 |
| Zhao C | 2016 | China | 126 (79/47) | I: 52.9 (43.9, 65.8) | HD | Aerobic exercise+escitalopram | Escitalopram | 18 weeks | Beck Depression Inventory-II | I: 29.87±17.24 | I: 22.31±16.26 | I: -7.56±16.77 |
|  |  |  |  | C: 54.1 (42.3, 68.7) |  |  |  |  |  | C: 35.85±16.53 | C: 27.29±17.44 | C: -8.56±17.00 ^5)^ |
| Carmack C | 1995 | USA | 48 (29/19) | 44.09 | HD | Aerobic exercise | Usual care | 10 weeks | Center for Epidemiologic Studies-Depression Scale | I: 11.9±11.8 | I: 6.8±8.2 | I: -5.1±10.5 |
|  |  |  |  |  |  |  |  |  |  | C: 8.0±7.4 | C: 5.0±5.0 | C: -3.0±6.5 |

^1)^ The study provides only the mean difference, and standard deviation is estimated as shown in **Table S5**.

^2)^ Data acquisition from Figure.

^3)^ Data acquisition by merging subgroups.

^4)^ The study reported two depression scales. After discussion, we chose the Beck Depression Inventory.

^5)^ The study reported depression levels in a multicategorical format, with mean and SD estimated as shown in **Table S6**.

Abbreviation: HD, hemodialysis; PD, peritoneal dialysis.

Table S5 Estimation of standard deviation for Carney et al.

| We use the following formula.  t = (x_1_ - x_2_) / sqrt(((n_1_-1)*s_1_^2 + (n_2_-1)*s_2_^2) / (n_1_+n_2_-2) * (1/n_1_ + 1/n_2_))  where  x_1_ = 3.4 (intervention group mean)  x_2_ = 9.7 (control group mean)  n_1_ = 10 (sample size of intervention group)  n_2_ = 7 (sample size of control group)  t = 2.49  df = 15  Assuming that the standard deviations of the two groups are equal, i.e., s1 = s2 = s, we can simplify this equation to  2.49 = (3.4 - 9.7) / (s * sqrt((1/10 + 1/7)))  Solving this equation, we get.  s = (-6.3) / (2.49 * sqrt(1/10 + 1/7)) = 2.27  Therefore, under this assumption, we estimate that the standard deviation of both groups after the intervention is 2.27. |
| --- |

Table S6 Estimation of standard deviation for Zhao C et al.

| In order to calculate the mean and standard deviation, we need to assume specific scores in each category. To simplify the calculations, we took the median of each category as the score for all subjects in that category. Namely,  Non-symptom (0-13 points): 6.5 points  Mild (14-19 points): 16.5 points  Moderate (20-28 points): 24 points  Severe (29-63 points): 46 points  At baseline.  Intervention group:  Mean = (13*6.5 + 9*16.5 + 8*24 + 33*46) / 63 = 29.87  Standard deviation = sqrt((13*(6.5-29.87)^2 + 9*(16.5-29.87)^2 + 8*(24-29.87)^2 + 33*(46-29.87)^2) / 62) = 17.24  Control Group:  Mean = (8*6.5 + 5*16.5 + 7*24 + 42*46) / 62 = 35.85  Standard deviation = sqrt((8*(6.5-35.85)^2 + 5*(16.5-35.85)^2 + 7*(24-35.85)^2 + 42*(46-35.85)^2) / 61) = 16.53  At endpoint.  Intervention Group.  Mean = (21*6.5 + 12*16.5 + 11*24 + 15*46) / 59 = 22.31  Standard deviation = sqrt((21*(6.5-22.31)^2 + 12*(16.5-22.31)^2 + 11*(24-22.31)^2 + 15*(46-22.31)^2) / 58) = 16.26  Control group.  Mean = (16*6.5 + 8*16.5 + 9*24 + 23*46) / 56 = 27.29  Standard deviation = sqrt((16*(6.5-27.29)^2 + 8*(16.5-27.29)^2 + 9*(24-27.29)^2 + 23*(46-27.29)^2) / 55) = 17.44  Thus, the Beck Depression Inventory scores for the intervention and control groups at baseline and endpoint were as follows.  Baseline.  Intervention group: 29.87 ± 17.24  Control group: 35.85±16.53  Endpoint.  Intervention group: 22.31±16.26  Control group: 27.29±17.44  Please note that these calculations are based on our assumptions about the scores in each category. |
| --- |

Table S7 Results of meta-regression.

| Variable | Estimate | SE | 95% CI | z/t | P | *I*^2^ | *R*^2^ |
| --- | --- | --- | --- | --- | --- | --- | --- |
| Age of participants in exercise group |  |  |  |  |  |  |  |
| Continuous | 0.011 | 0.021 | -0.032, 0.054 | 0.511 | 0.615 | 86.70% | 0.00% |
| Categorical (< 60 vs. >=60) | -0.021 | 0.394 | -0.842, 0.799 | -0.054 | 0.957 | 86.59% | 0.00% |
| Male proportion |  |  |  |  |  |  |  |
| Continuous | -0.004 | 1.432 | -2.982, 2.974 | -0.003 | 0.998 | 86.41% | 0.00% |
| Categorical (<50% vs. >=50%) | 0.241 | 0.440 | -0.673, 1.155 | 0.549 | 0.589 | 86.15% | 0.00% |
| Duration of intervention |  |  |  |  |  |  |  |
| Continuous | -0.006 | 0.014 | -0.036, 0.023 | -0.461 | 0.649 | 86.81% | 0.00% |
| Categorical (<=12 vs. >12) | -0.398 | 0.314 | -1.051, 0.255 | -1.267 | 0.219 | 85.94% | 0.00% |
| Total sample |  |  |  |  |  |  |  |
| Continuous | 0.004 | 0.003 | -0.003, 0.011 | 1.247 | 0.226 | 85.25% | 0.01% |
| Categorical (<=60 vs. >60) | 0.257 | 0.324 | -0.417, 0.931 | 0.793 | 0.437 | 86.60% | 0.00% |
| Exercise type |  |  |  |  |  | 86.33% | 0.00% |
| Aerobic exercise | Ref. |  |  |  |  |  |  |
| Resistance training | 0.293 | 0.584 | -0.925, 1.510 | 0.501 | 0.622 |  |  |
| Combined exercise | -0.154 | 0.351 | -0.885, 0.577 | -0.440 | 0.665 |  |  |
| Publication year |  |  |  |  |  |  |  |
| Continuous | 0.009 | 0.019 | -0.030, 0.048 | 0.460 | 0.650 | 87.01% | 0.00% |
| Categorical (before 2010 vs. 2010 and after) | -0.111 | 0.362 | -0.864, 0.643 | -0.305 | 0.763 | 86.89% | 0.00% |
| Disease stage |  |  |  |  |  | 85.70% | 0.00% |
| Hemodialysis | Ref. |  |  |  |  |  |  |
| Predialysis | 0.495 | 0.468 | -0.482, 1.472 | 1.057 | 0.303 |  |  |
| Peritoneal dialysis | -0.302 | 0.755 | -1.876, 1.272 | -0.401 | 0.693 |  |  |

Figure S1 Risk of bias for including studies.


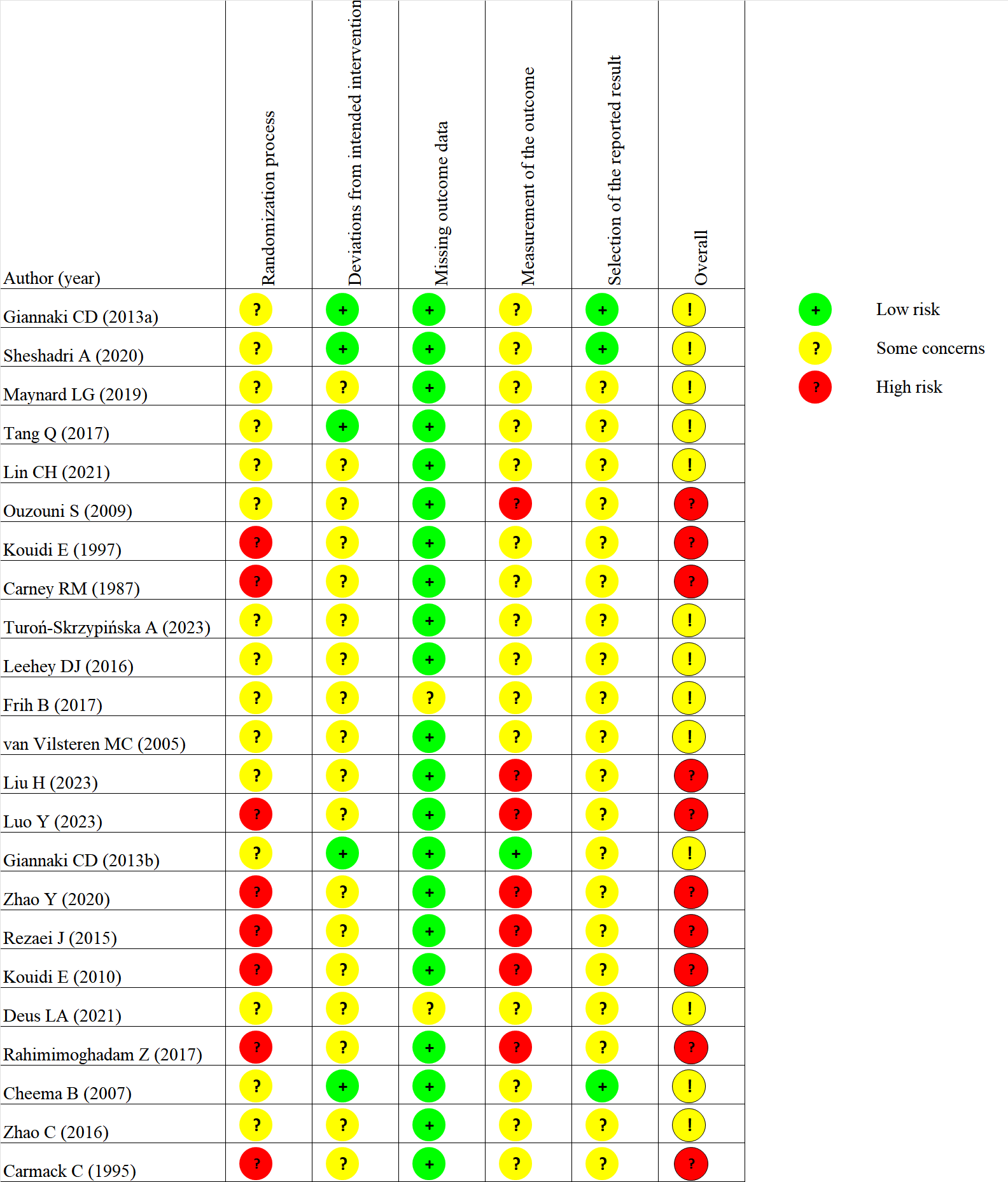


Figure S2 Sensitivity analysis excluding "outliers studies"


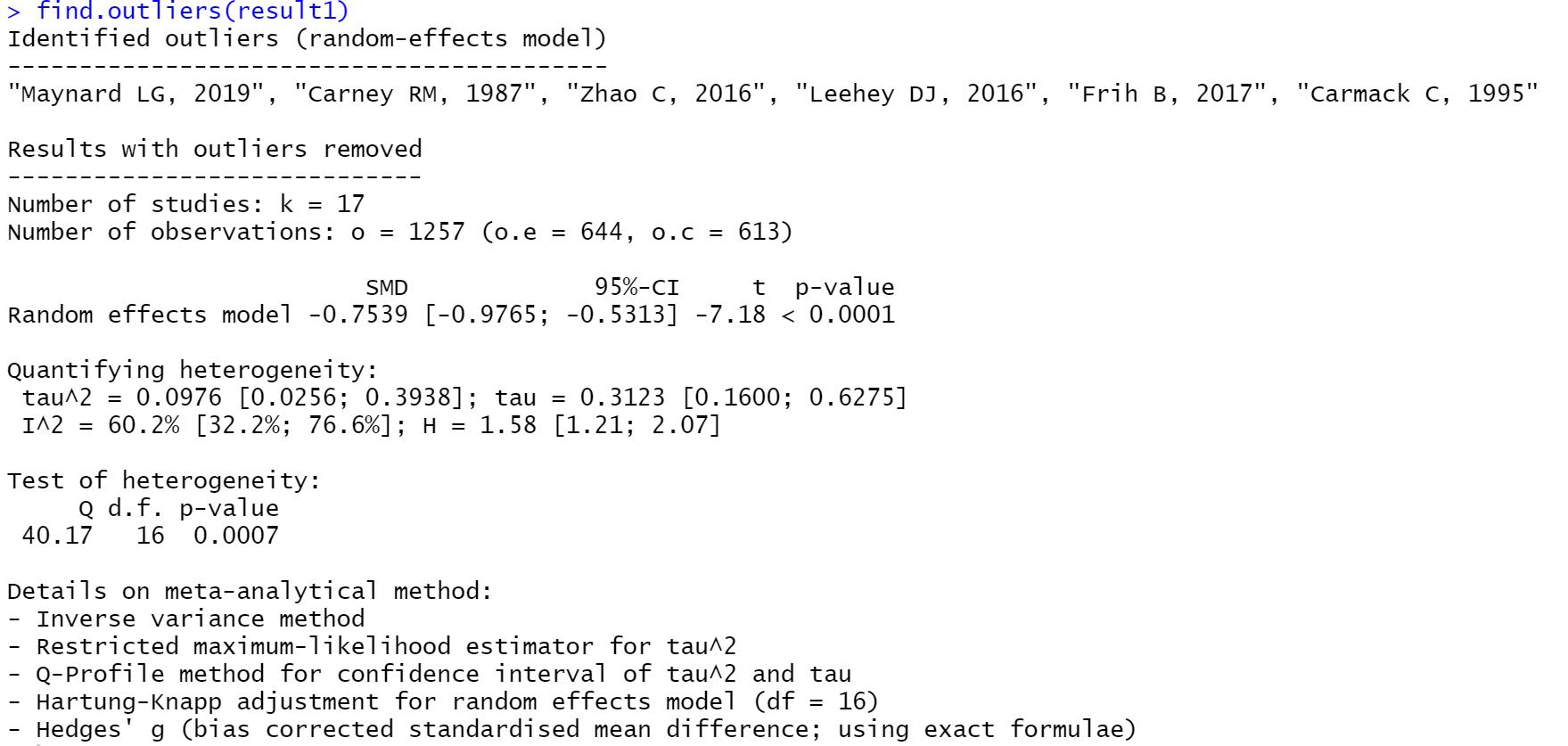


Figure S3 Sensitivity analysis excluding "study on data estimation".


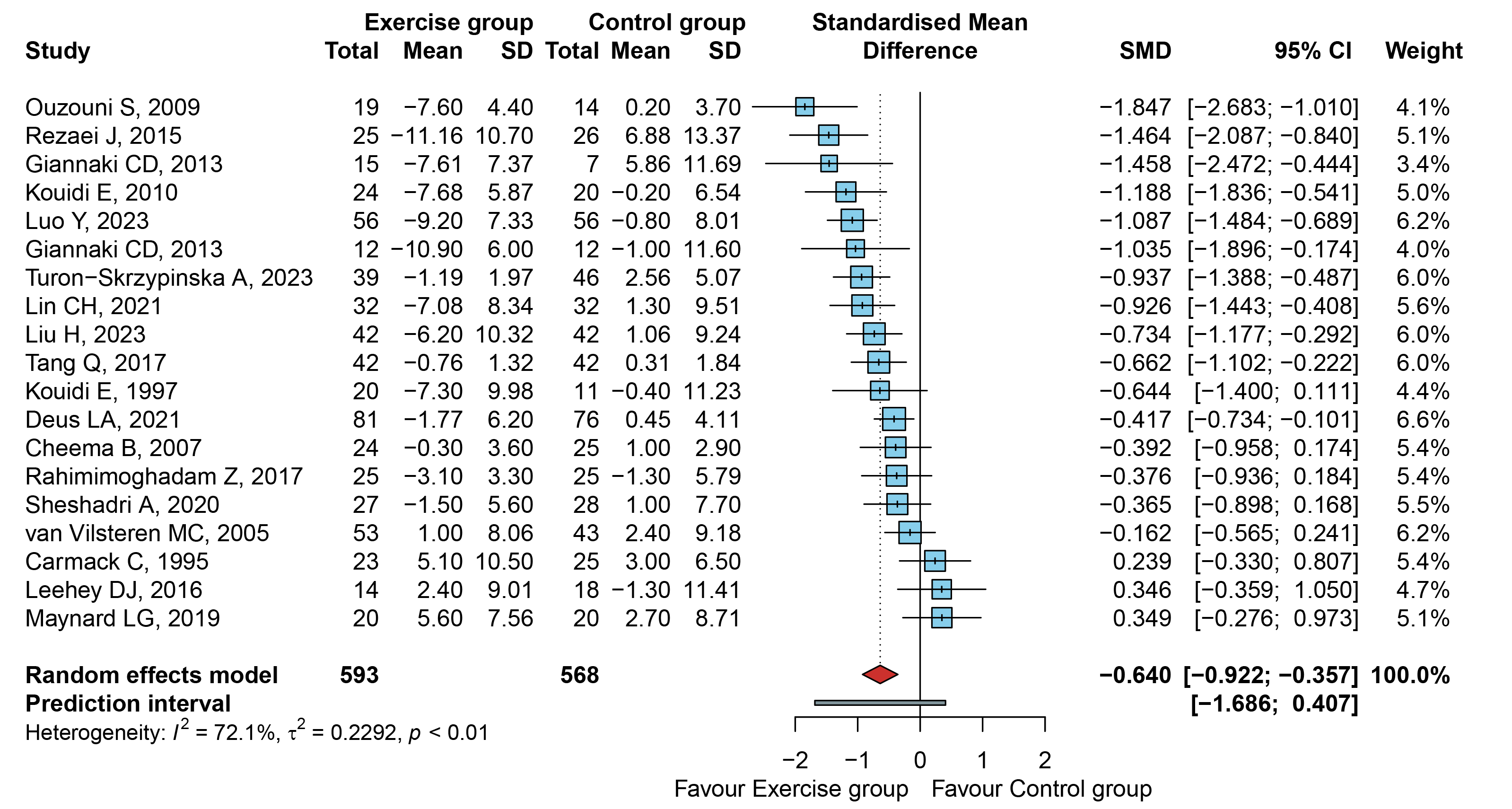


Figure S4 Sensitivity analysis excluding "studies published before 2010".


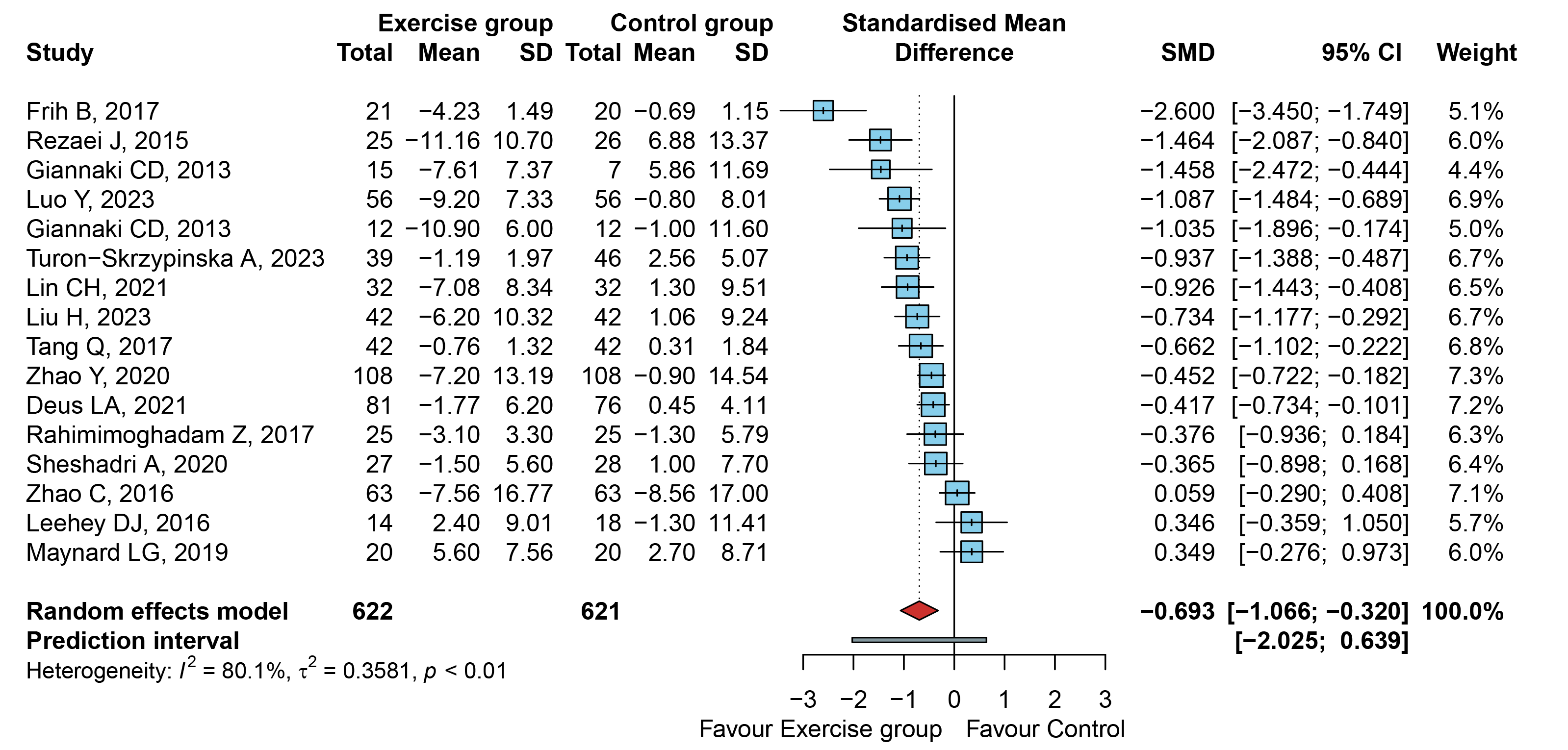


Figure S5 Sensitivity analysis based on "leave one out" approach.

**
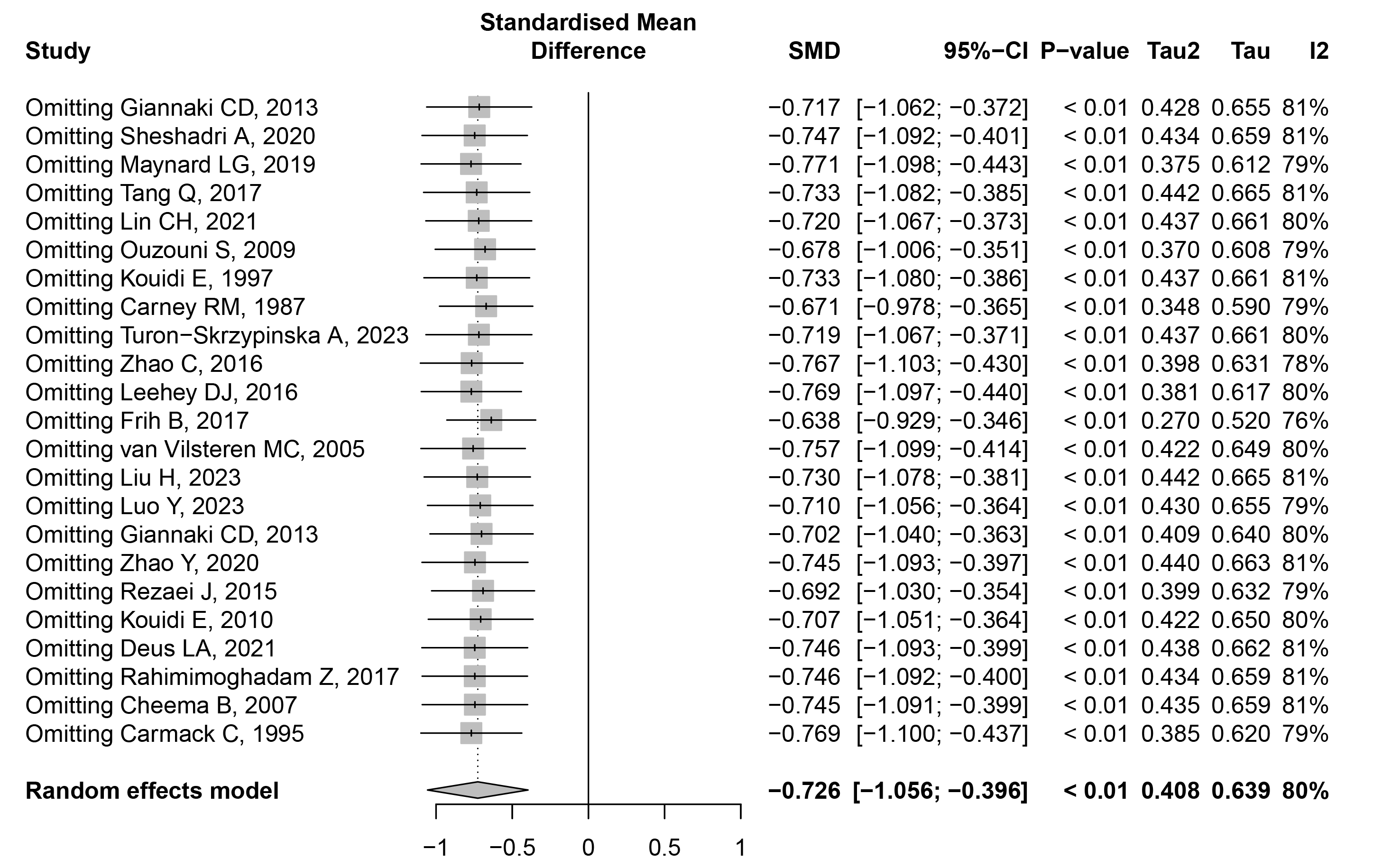
**

Figure S6 Contour-enhanced funnel plot.


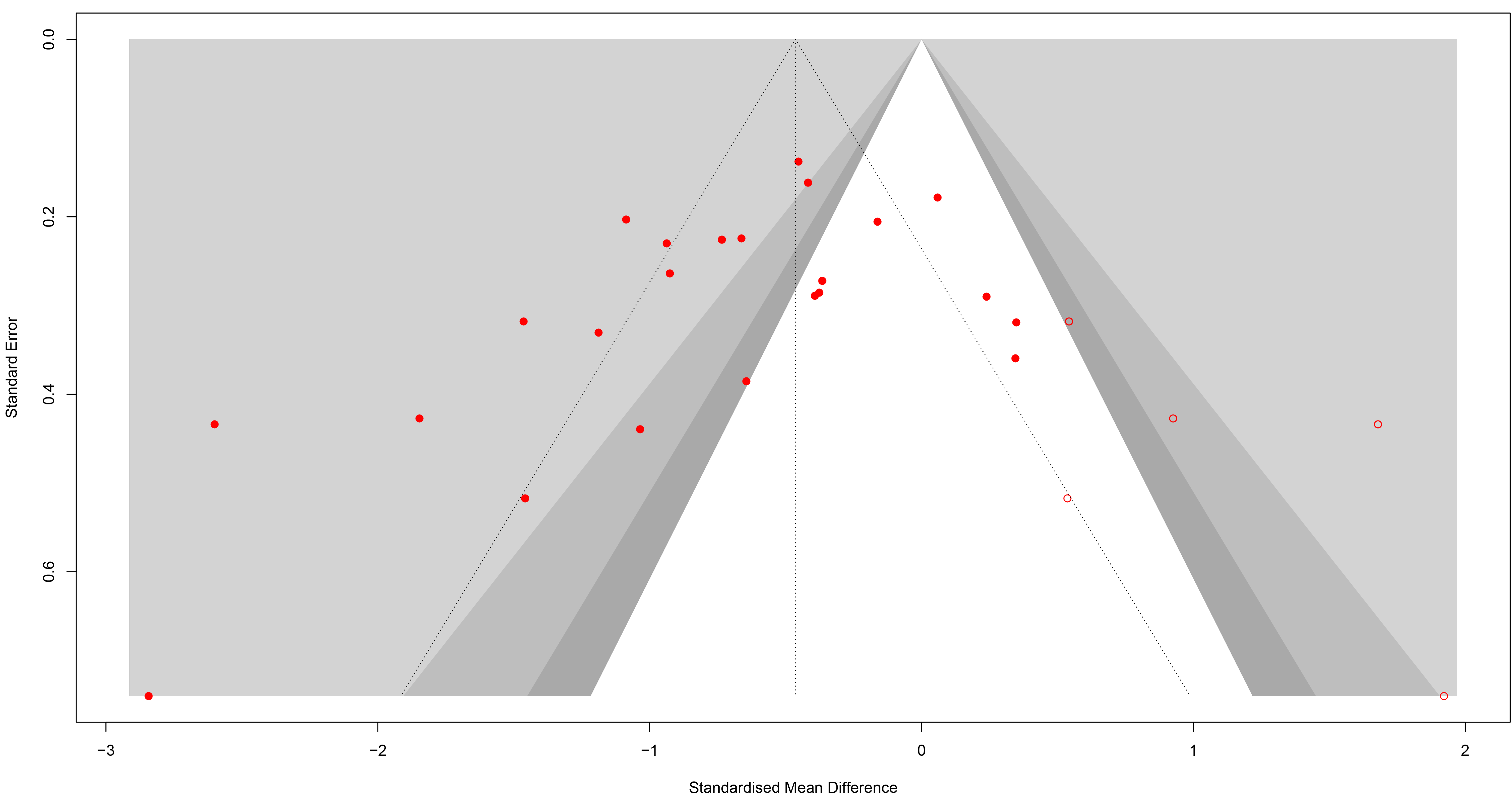


The supplemental studies are distributed in the area of no statistical significance (white area), indicating the presence of unpublished studies that do not have statistical significance. Therefore, this meta-analysis exhibits a certain degree of publication bias.


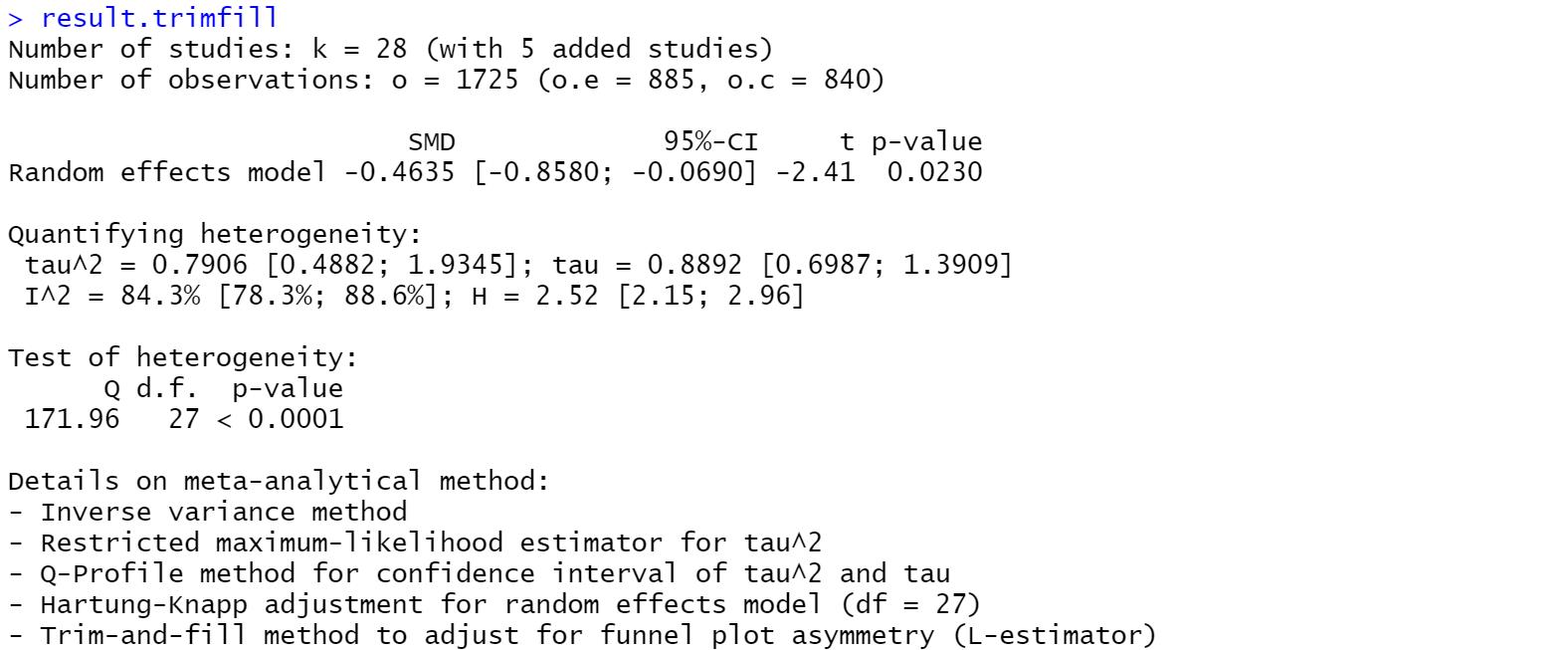

Supplement: Supplemental Material [file IRNF_A_2436105_SM6894.docx]
